# Colorimetric Nanobiosensor Design for Determining Oxidase Enzyme Substrates in Food and Biological Samples

**DOI:** 10.1021/acsomega.2c06053

**Published:** 2022-11-23

**Authors:** Aslı Neslihan Avan, Sema Demirci-Çekiç, Reşat Apak

**Affiliations:** † Department of Chemistry, Faculty of Engineering, Istanbul University-Cerrahpasa, Avcilar, 34320Istanbul, Turkiye; ‡ Department of Chemistry, Institute of Graduate Studies, Istanbul University-Cerrahpasa, 34320Istanbul, Turkiye; § Turkish Academy of Sciences (TUBA), Vedat Dalokay St. No. 112, 06670Cankaya, Ankara, Turkiye

## Abstract

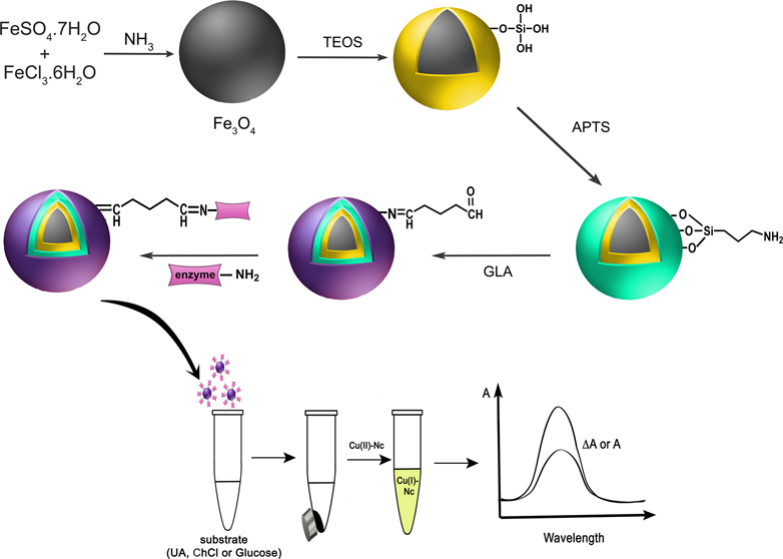

Biological enzymes have high catalytic activity and unique substrate selectivity; their immobilization may provide cost reduction and reusability. Using magnetic nanoparticles (MNPs) as support materials for enzymes ensures easy separation from reaction media by an external magnetic field. Thus, MNPs were prepared by the coprecipitation method, coated with silanol groups, then −NH_2_-functionalized, and finally activated with glutaraldehyde. Finally, three different oxidase enzymes, i.e., uricase, glucose oxidase, and choline oxidase, were separately immobilized on their surfaces by covalent attachment. Hence, colorimetric nanobiosensors for the determination of three biologically important substrates, i.e., uric acid (UA), glucose (Glu), and choline (Ch), were developed. Hydrogen peroxide liberated from enzyme–substrate reactions was determined by the cupric ion reducing antioxidant capacity (CUPRAC) reagent, bis-neocuproine copper(II) chelate. In addition, UA-free total antioxidant capacity could also be measured via the developed system. Kinetic investigations were carried out for these nanobiosensors to enable the calculation of their Michaelis constants (*K*_m_), revealing no affinity loss for the substrate as a result of immobilization. Enzyme-immobilized MNPs could be reused at least five times. The linear concentration ranges were 5.43–65.22 μM for UA, 11.1–111.1 μM for Glu, and 2.78–44.4 μM for Ch, and the limit of detection (LOD) values with the same order were 0.34, 0.59, and 0.20 μM, respectively. Besides phenolic and thiol-type antioxidants, UA could be determined with an error range of 0.18–4.87%. The method is based on a clear redox reaction with a definite stoichiometry for the enzymatically generated H_2_O_2_ (which minimizes chemical deviations from Beer’s law of optical absorbances), and it was successfully applied to the determination of Glu and UA in fetal bovine serum and Ch in infant formula as real samples.

## 1 Introduction

Biosensors have recognition sites for biomolecules such as enzymes, DNA, and antibodies. They can provide selective and sensitive detection for vital biomolecules serving the diagnosis of different diseases.^1^ In recent years, nanoscale sensors having high surface area and superior catalytic properties have been developed.^2^ Among different biorecognition elements, enzymes offer unique selectivity to the tested analyte. Nanozymes discovered in the last few decades can hardly compete with enzymes due to their lower specificity and turnover ratios. However, enzymes have poor stability, and complications faced with their use and separation during in vitro studies restrict their usage and increase the cost. But anchoring enzymes on solid supports by immobilization helps to overcome these drawbacks.^1^ Here, another difficulty appears in the separation of immobilized enzymes from the reaction medium. A centrifugation operation serving this purpose may cause problems because of the small size of nanoparticles (NPs).^3^ Among the different solid supports that can be used for separation, magnetic nanoparticles (MNPs) have a special place with their easy separation by means of an external magnetic field. In addition to their easy separation, MNPs also have other advantages such as a high specific surface area^4^ and electrostatic ability to bind probes, thereby making Fe_3_O_4_ MNPs eligible as solid supports for enzyme immobilization. To synthesize Fe_3_O_4_ MNPs, several techniques were offered in the literature such as coprecipitation, microemulsion formation, thermal decomposition, and solvothermal processes.^3^ The large surface area and high surface energies of Fe_3_O_4_ nanoparticles endow them with a tendency to aggregate. They can also lose their magnetism and dispersibility easily by air oxidation.^5^ Therefore, surface functionalization is frequently used to prevent the aggregation and oxidation of MNPs.^4^

Three different oxidase enzymes, namely, glucose oxidase (GOx), choline oxidase (ChOx), and uricase (UOx), were immobilized separately on Fe_3_O_4_ nanoparticles synthesized by coprecipitation as described by Chang et al.^6^ Before immobilization, MNPs were modified by tetraethyl orthosilicate (TEOS), 3-aminopropyltriethoxysilane (APTS), and glutaraldehyde (GLA) in this order, followed by covalent attachment of enzymes. Then, the immobilized oxidase enzymes reacted with their substrates to generate H_2_O_2_, subsequently determined by the simple and effective CUPRAC colorimetric method, efficiently responding to both H_2_O_2_ and uric acid.

All three oxidase substrates, namely, glucose (Glu), choline chloride (ChCl), and uric acid (UA), were biologically important compounds for which development of detection methods remains an analytical challenge. Glucose is important for the food industry and biochemistry (i.e., the most important carbohydrate fuel in the body). Precise and accurate measurement of blood glucose helps to diagnose and manage diabetes disease for minimizing its complications.^2^ Glucose detection strategies comprise colorimetric, fluorometric, chemiluminescent, and electrochemical methods and have recently been complemented with nanosensing technologies.^7^ The idea of enzymatic determination of glucose (still effective today) dates back to the early 1960s.^8,9^ The second analyte, choline (Ch), is not only one of the main physiological components in mammals but is an essential nutrient whose daily intake is highly recommended due to its participation in the synthesis of the neurotransmitter acetylcholine.^10^ The third substrate, UA, is an important antioxidant (AOx) that makes a significant contribution to plasma total antioxidant capacity (TAC).^11^ However, its elevated levels in the blood may cause hyperuricemia, which may lead to a painful type of arthritis called gout and, secondarily, to heart and kidney disease.^12^ Literature studies regarding UA determination generally involve colorimetry, chromatography, chemiluminescence, and fluorometry.^13^ Colorimetric methods offer certain advantages to chromatography and electrochemistry such as ease of operation, low cost, practicality, etc.^14^ However, especially in complex matrices such as biological samples, there can be so many interferences that may adversely affect colorimetric methods. In this regard, the use of UOx can ensure selectivity, since the enzyme substrate (UA) is selectively oxidized by UOx to release H_2_O_2_ quantitatively, which can in turn be determined by another enzyme, i.e., horseradish peroxidase (HRP).^15^

Colorimetric biosensors have a special importance among other methods because of their operation ease, rapid response, and relatively low cost. In addition, the generated colored product can be observed even by the naked eye.^16^ An enzymatic colorimetric method is presented in the study. Unlike many other studies in the literature using *o*-phenylenediamine, 4-aminoantipyrine (4-AAP, in the presence of phenol), 2,2-azino-bis(3-ethylbenzothiazoline-6-sulfonic acid (ABTS), and 3,3',5,5'-tetramethylbenzidine (TMB) as peroxidase substrates,^17^ the CUPRAC reagent (Cu(II)-Nc) was used as the chromogenic agent to oxidize H_2_O_2_ to O_2_ by a net stoichiometric reaction, thereby ensuring obedience to Beer’s law for optical absorption.

To the best of our knowledge, the presented study differs from its counterparts in terms of shedding light on the determination of three important biological compounds (as enzyme substrates) with the help of three different oxidase enzymes. The liberated hydrogen peroxide from enzyme–substrate reactions was determined with the robust CUPRAC method instead of peroxidase assays widely used for H_2_O_2_ estimation but adversely affected by peroxidase inhibitors. The CUPRAC reagent, bis-neocuproine copper(II) complex, is one of the rare oxidants capable of fully oxidizing H_2_O_2_ to molecular O_2_ (as a single product) without an activator. In addition, the activities of free and immobilized enzymes were compared through kinetic examinations, where immobilized enzymes could be repeatedly used with tolerable loss in activity. Furthermore, real sample applications showed that the proposed method is suitable for substrate determination in complex matrices such as biological and food samples.

## 2 Materials and Methods

The preparation of the solutions and reagents used in the experiments is given in Section S.I.1 to reduce the volume of the manuscript.

### 2.1 Materials

Chemicals that were used in the study were purchased from different sources: CuCl_2_·2H_2_O, glutaraldehyde (GLA, 25 wt % in water), tri-sodium citrate 5,5-hydrate, FeCl_3_·6H_2_O, FeSO_4_·7H_2_O, d-(+)-glucose (Glu), phenol, Na_2_HPO_4_·2H_2_O, and NaH_2_PO_4_·2H_2_O were purchased from Merck; uric acid (UA), (+)-catechin hydrate (CAT), bilirubin (Bil), caffeic acid (CFA), fetal bovine serum (FBS), quercetin (QR), catalase from bovine liver, uricase (UOx) from *Candida* sp., glucose oxidase (GOx) from *Aspergillus niger*, choline oxidase (ChOx) from *Alcaligenes* sp., peroxidase from horseradish (HRP), choline chloride (ChCl), glycine, neocuproine (Nc) hydrochloride hydrate, and trizma base were purchased from Sigma; (3-aminopropyl)triethoxysilane (APTS), *N*-acetyl-l-cysteine (NAC), tetraethyl orthosilicate (TEOS), urea, 4-aminoantipyrine (4-AAP), ascorbic acid (AA), gallic acid monohydrate (GA), l-glutathione reduced (GSH) were from Sigma-Aldrich; and *trans*-ferulic acid (FA), (*R*)-(+)-6-hydroxy-2,5,7,8-tetramethylchroman-2-carboxylic acid (trolox) (TR), and l-cysteine (CYS) were from Aldrich.

### 2.2 Apparatus

A Varian CARY 100 UV–vis spectrophotometer (Mulgrave, Victoria, Australia) was used for spectrophotometric measurements. High performance liquid chromatography (HPLC) instrument consisting of a Waters 1525 binary HPLC pump, an in-line degasser, and a Waters 2998 PDA detector (Milford, Massachusetts) was used for HPLC determinations. In the study, a ZORBAX eclipse C18 (250 × 4.6 mm, 5 μm) reverse-phase column was used as the stationary phase. The characterization of the synthesized nanoparticles was performed by scanning transmission electron microscopy (STEM)–energy-dispersive X-ray spectrometry (EDS) analysis (Thermo Scientific Quattro FEG SEM), Fourier transform infrared spectroscopy (FTIR, Agilent Carry 630 FTIR-ATR), and X-ray photoelectron spectroscopy (XPS) conducted using a K-Alpha spectrometer (Thermo Fisher) employing a monochromated Al Kα X-ray source (*h*ν = 14,686.6 eV).

### 2.3 Synthesis, Modification, and Enzyme Immobilization for Fe_3_O_4_ MNPs

Fe_3_O_4_ MNPs were synthesized with the coprecipitation method as described by Chang et al.^6^ Then, the surface of the obtained Fe_3_O_4_ MNPs was coated with silanol according to the Stöber method.^18^ For this purpose, 0.2 g of Fe_3_O_4_ was mixed with 100 mL of ethanol, 20 mL of water, 1.0 mL of concentrated NH_3_, and 0.2 mL of TEOS at room temperature for 6 h. Then, it was washed several times with water and ethanol and dried at 70 °C. After 0.5 g of the prepared Fe_3_O_4_/SiO_2_ was dispersed in 50 mL of ethanol in an ultrasonic bath for 1 h, 4.2 g of APTS was added and mixed at room temperature for 20 h. After washing several times with water, the prepared Fe_3_O_4_/SiO_2_/APTS was dried at 70 °C.^19^

A volume of 200 μL of GLA and 4.8 mL of pH 7.0 phosphate buffer were added to 0.1 g of Fe_3_O_4_/SiO_2_/APTS and mixed for 1.5 h to activate the modified MNPs. In this way, MNPs prepared for enzyme immobilization were collected with the aid of a magnet and washed several times with distilled water.

Three different oxidase enzymes, namely, GOx, ChOx, and UOx, were separately immobilized on the modified MNPs. For this purpose, enzyme solutions were prepared at different concentrations using different pH buffers, i.e., UOx (0.4 mg mL^–1^) in 0.1 M pH 8.5 phosphate buffer solution (PBS), GOx (2.0 mg mL^–1^) in pH 7.0 PBS (0.1 M), and ChOx (0.4 mg mL^–1^) in (0.2 M) pH 6.0 acetic acid/sodium acetate buffer solution (ABS). Then, 5.0 mL of each enzyme solution was added to 0.1 g of modified and activated MNPs (Fe_3_O_4_/SiO_2_/APTS/GLA) separately. To ensure complete binding of the tested enzymes, GOx + MNPs and ChOx + MNPs were mixed for 30 min at room temperature with the aid of a rotator. For UOx, MNPs and enzyme solution were kept at 4 °C for 5 h. The enzyme-attached MNPs (UOx@MNPs, GOx@MNPs, and ChOx@MNPs) were washed with the indicated buffer solution and distilled water a few times and finally dispersed in 5.0 mL of distilled water for use in the determination of the corresponding substrates (i.e., UA, Glu, and Ch).

### 2.4 Determination of Glu, ChCl, UA, and UA-Free TAC by the Proposed Method

It can be said that the proposed method consists of two main parts: first, H_2_O_2_ was generated by means of the reaction between related MNPs-immobilized oxidase and its substrate, and second, the generated H_2_O_2_ was determined by the CUPRAC method.^20^ Here, while the first part contains some differences, the second step was the same for Glu and ChCl determination.

#### 2.4.1 Proposed Procedure for Glu Quantification

A volume of 0.5 mL of pH 7.0 NH_4_Ac buffer, *x* mL of Glu, (0.8 – *x*) mL of distilled water, and 200 μL of GOx@MNPs were mixed in this order, and the reaction mixture was mixed for 15 min using a rotator. Then, after separation of GOx@MNPs with a magnet, the CUPRAC reagent mixture (1.0 mL of 1.0 × 10^–2^ M CuCl_2_ + 1.0 mL of 7.5 × 10^–3^ M Nc + 1.0 mL 1.0 M NH_4_Ac) was added to the same test tube. The absorbance values were measured after incubation for 30 min at room temperature.

#### 2.4.2 Proposed Procedure for ChCl Quantification

A volume of 0.5 mL of 0.2 M NH_3_/NH_4_Cl buffer solution at pH 9.0, *x* mL of ChCl, (0.85 – *x*) mL of distilled water, and 150 μL of ChOx@MNPs were mixed in this order. After the reaction mixture was mixed with a rotator for 20 min, nanoparticles were removed by means of a magnet. After separation of ChOx@MNPs, the CUPRAC reagent mixture was added, and the operation was continued as stated above.

#### 2.4.3 Determination of UA and UA-Free TAC

Half a milliliter (0.5 mL) of pH 8.5 PBS (0.1 M), *x* mL of UA (or UA-containing antioxidant sample), (0.9 – *x*) mL of H_2_O, 100 μL of ethanol (EtOH), 50 μL of (0.1 mg mL^–1^) catalase, and 50 μL of UOx@MNPs were mixed in this order; the reaction mixture was incubated at 37 °C for 15 min. Then, UOx@MNPs was separated by a magnet. Then, the CUPRAC reagent mixture (1.0 mL of 1.0 × 10^–2^ M CuCl_2_ + 1.0 mL of 7.5 × 10^–3^ M Nc + 1.0 mL of pH 7.0 urea buffer) was added, and the operation was continued as stated above.

It should be noted here that, unlike Glu and ChCl determinations, catalase was included in the proposed method for the determination of UA and UA-free total antioxidant capacity (TAC). The CUPRAC reagent reacts with antioxidant compounds as well as with H_2_O_2_. In an AOx mixture, only UA reacts with immobilized UOx to form H_2_O_2_. As a result of the decomposition of H_2_O_2_ with catalase added to the system, the contribution of UA to TAC was eliminated. When the CUPRAC method was applied to an AOx mixture before and after treatment with immobilized UOx, the difference between the two CUPRAC absorbances indicated UA concentration.

### 2.5 Drawing the Calibration Graphs for the Tested Substrates by the Proposed Method

The appropriate volumes taken from the substrate solutions were subjected to the procedure described in Section S.I.3 to establish the calibration curves.

### 2.6 UA Determination in AOx Mixtures by the Proposed UOx@MNPs Method

A synthetic AOx mixture containing UA was treated with the CUPRAC reagent before and after treatment with immobilized UOx. In addition, UA determination was performed in another mixture consisting of common serum AOxs (Bil, AA, GSH), as explained in Section S.I.4.

### 2.7 Chromatographic UA Determination

To compare the results obtained by the proposed method, a reverse-phase HPLC method was used as the standard verification method. For testing the amount of UA in the FBS, the method described earlier by George et al.^21^ was applied with a few modifications. The details were given in Section S.I.5.

### 2.8 Application of the Proposed Method to the Selected Real Samples

For testing the proposed procedure in complex sample matrices, while FBS was used as the real sample for Glu and UA determinations, a commercially available infant formula was chosen for ChCl. In addition, the method of standard additions was employed for the three analytes to calculate recoveries. To verify the results, the obtained values for Glu and ChCl were compared to the declared values by the producers of tested real samples. The UA value was also compared with the finding of the standard HPLC method. The proposed methods are explained in detail in Section. S.I.6.

### 2.9 Investigation of Enzyme Kinetics

#### 2.9.1 Determination of Enzyme Kinetics for GOx and ChOx

To investigate enzyme kinetics, Michaelis constant (*K*_m_) and maximum velocity of the enzymatically catalyzed reaction (*V*_max_) values were calculated for free and immobilized enzymes. To determine the activity of free and immobilized GOx and ChOx, the formation rate of enzymatically generated H_2_O_2_ was monitored at 25 ± 0.1 °C. For this purpose, an HRP-catalyzed reaction in the presence of phenol and 4-AAP was applied (Figure S1).^22^ The details of the method are given in Section S.I.7.1.

To determine the activity of the covalently attached enzyme, the GOx concentration was prepared to be the same as that of the free enzyme. For this purpose, 2.0 mg of GOx and 1.0 mL of PBS (at pH 7.0) were added to 0.02 g of Fe_3_O_4_/SiO_2_/APTS/GLA and incubated for 30 min, then washed with PBS (pH 7.0), and dispersed in 2.0 mL of distilled water; 10 μL was taken from it, and its activity was measured.

Similarly, for ChOx, 0.1 mL of ChOx (24 U mL^–1^) and 0.9 mL of ABS (at pH 6.0) were added to 0.01 g of Fe_3_O_4_/SiO_2_/APTS/GLA and incubated for 30 min. After incubation, it was washed with water and dispersed in 0.5 mL of water, and its activity was determined by taking 50 μL from it.

#### 2.9.2 Determination of Enzyme Kinetics for UOx

Uricase activity was determined by measuring the decrease in substrate concentration, i.e., using the UV spectrometric determination of UA.^23^ To determine the *K*_m_ and *V*_max_ values of free and immobilized enzymes, 20 μL of free UOx at a concentration of 0.4 mg mL^–1^ and a suitable amount of MNPs containing the same amount of covalently bound UOx were treated with different concentrations of UA varying between 3.33 × 10^–5^ and 1.67 × 10^–4^ M at 25 ± 0.1 °C. To determine UA concentration, the absorbance values were measured at 290 nm, and the decrease in absorbance at the end of the 5th min was examined.

### 2.10 Investigation of Stability and Reusability of MNPs-Attached Enzymes

The enzyme-attached MNPs were kept in a sealed glass bottle at 4 °C, and to investigate their stability, the tests for determining their substrates were repeated at different times between 1 and 60 days. For this purpose, 65.2 μM UA, 100 μM Glu, and 33.3 μM ChCl were used. In addition, the same MNPs-attached enzymes were used several times to oxidize their substrates. Immobilized enzymes were tested for two different concentrations of the substrates; the enzyme-attached MNPs were collected with a magnet, washed with distilled water, and repeatedly used five times.

To perform intraday repeatability and interday reproducibility experiments, the related methods were applied to 65.2 μM UA, 88.9 μM Glu, and 33.3 μM ChCl five times a day and on five consecutive days, and the % relative standard deviation (RSD) values were calculated.

### 2.11 Examination of the Effects of Experimental Conditions on the Immobilized Enzymes

As is known, biological enzymes are not stable in harsh laboratory conditions. Therefore, the stability of immobilized enzymes against temperature, different pH values, and some commonly used solvents was investigated. The details of the experiments are given in Section S.I.2. (The obtained results were shown in Figures S2–S4.)

## 3 Results and Discussion

### 3.1 Synthesis and Characterization of Enzyme-Attached MNPs

The surface of Fe_3_O_4_ MNPs, synthesized according to the method of Chang et al.,^6^ was coated with silanol groups in accordance with the Stöber method using TEOS.^19^ Then, by the addition of APTS, hydroxyl groups of hydrolyzed APTS reacted with silanol groups of the silica layer to obtain an organosilane layer. As a result, the hydrophilic nature of the silica surface became slightly hydrophobic after organo-functionalization. Although the specific surface area of silica decreased after modification with APTS, the NH_2_ groups on the surface of Fe_3_O_4_/SiO_2_/APTS could act as a binder for organic moieties. Finally, the covalent binding of the enzyme was ensured by the formation of −CH=N– as a result of the reaction between the terminal −COH group of GLA and the N atom of the enzymes.

To investigate the structure of the synthesized, modified, and enzyme-attached MNPs, scanning transmission electron microscopy (STEM)–energy-dispersive X-ray spectrometry (EDS) analysis was used. Another characterization study was carried out by Fourier transform infrared spectroscopy (FTIR).

**Figure 1:**
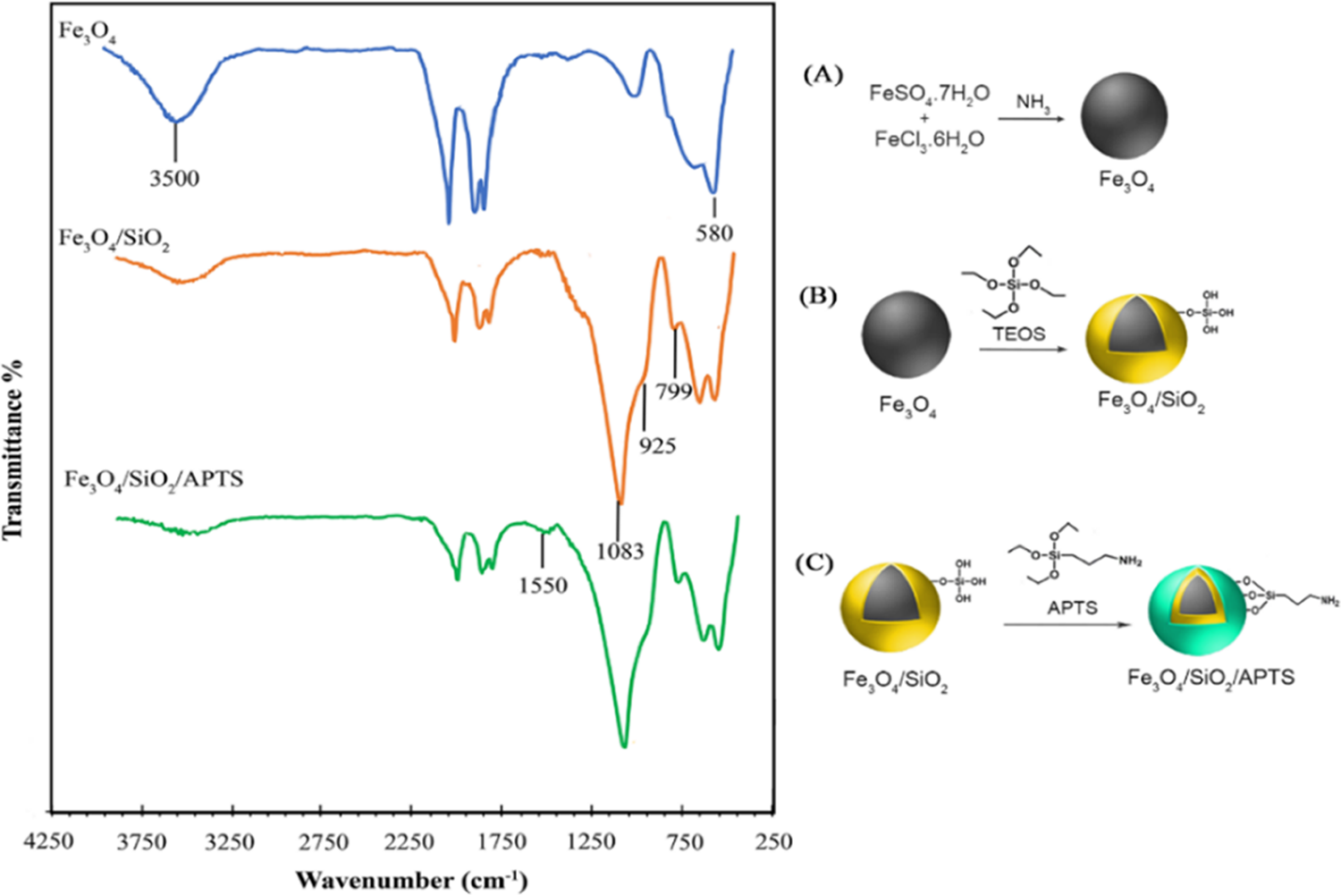
FTIR spectra for (A) Fe_3_O_4_-MNPs, (B) Fe_3_O_4_/SiO_2_: SiO_2_-modified MNPs made by the tetraethyl orthosilicate (TEOS) reaction, and (C) Fe_3_O_4_/SiO_2_/APTS: APTS-modified Fe_3_O_4_/SiO_2_.

Here, while STEM images were taken for Fe_3_O_4_ MNPs and UOx-attached MNPs (the final form of the synthesized nanobiosensor), FTIR analysis was made for different stages of the nanobiosensor fabrication. These stages were as follows: intact Fe_3_O_4_ MNPs (Figure 1A), SiO_2_-modified MNPs (called Fe_3_O_4_/SiO_2_, Figure 1B), and APTS-modified Fe_3_O_4_/SiO_2_ (called Fe_3_O_4_/SiO_2_/APTS, Figure 1C).

The FTIR spectra shown in Figure 1 showed that the absorption peaks seen around 580 and 3500 cm^–1^ in the spectrum of Fe_3_O_4_ belong to the Fe–O and O–H stretching vibrations. The peaks seen at 1083 and 799 cm^–1^ in the spectrum of Fe_3_O_4_/SiO_2_-modified MNPs can be associated with nonsymmetrical and symmetric linear stretching vibrations of the Si–O–Si bond. The absorption peak of the bending vibration of Si–OH was observed at 925 cm^–1^. The N–H vibrational absorption peak of the free NH_2_ group in the Fe_3_O_4_/SiO_2_/APTS spectrum is observed at 1550 cm^–1^, indicating that APTS is attached to the SiO_2_ surface.

**Figure 2:**
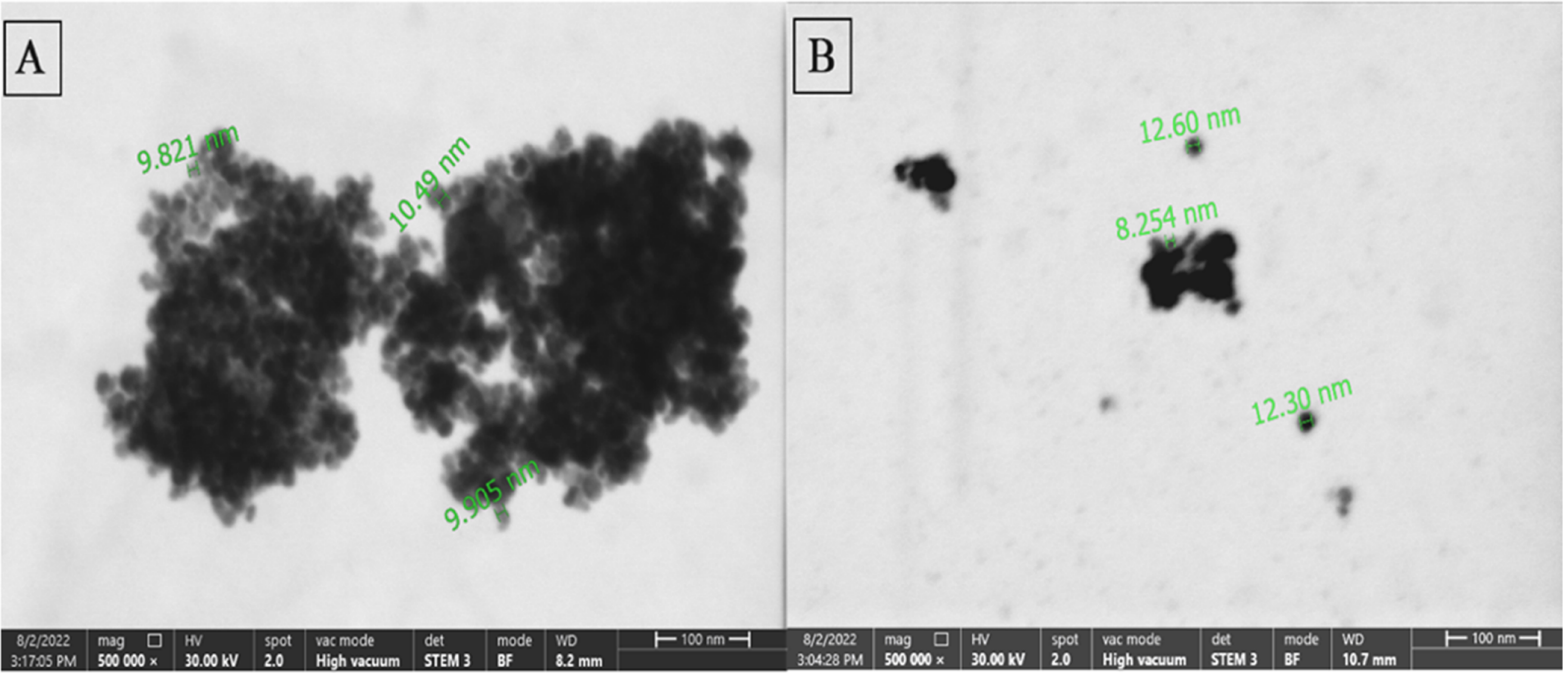
Different STEM images (A and B) taken for synthesized bare Fe_3_O_4_ MNPs (before modification).

**Figure 3:**
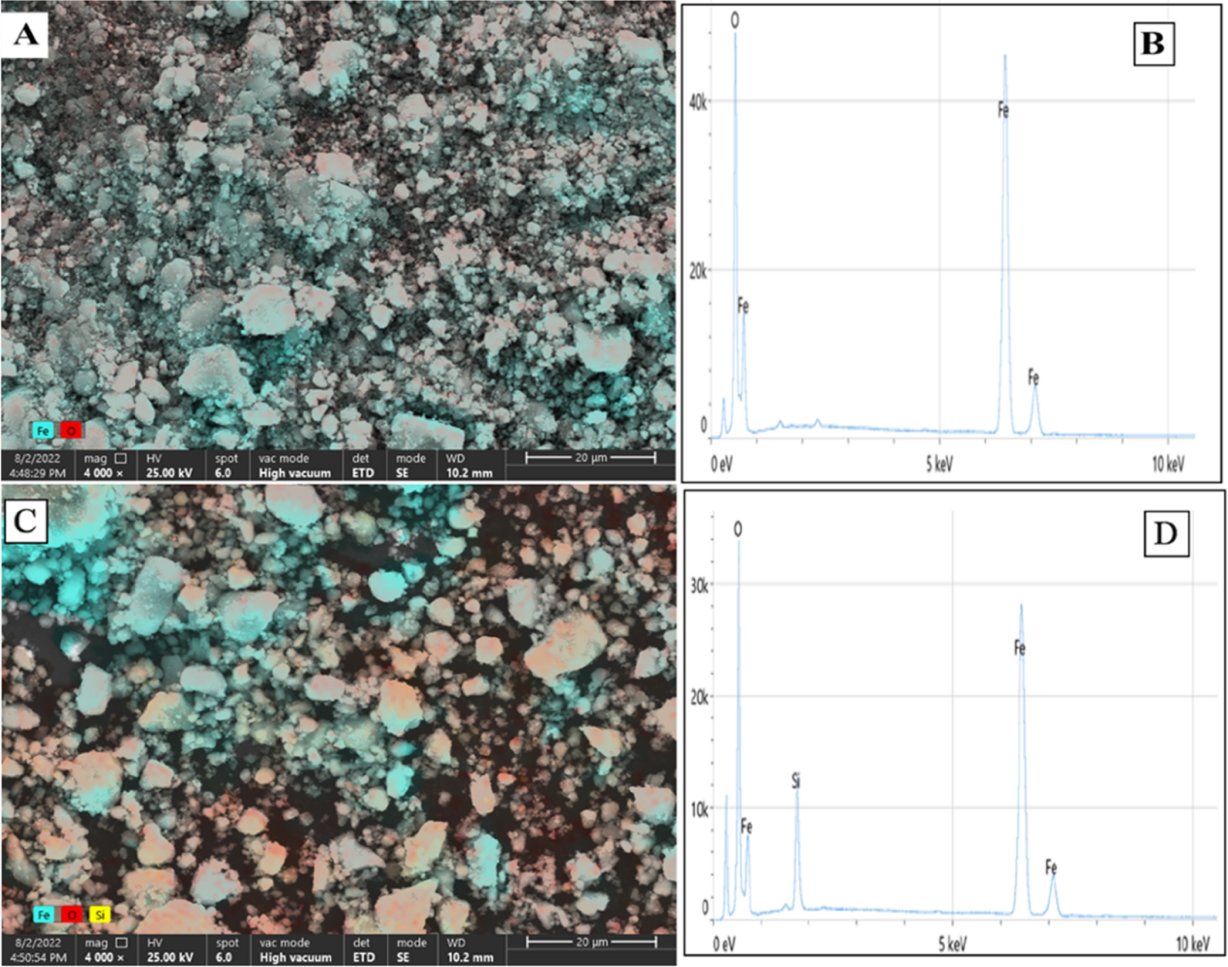
EDS spectra and color STEM images of bare Fe_3_O_4_ MNPs (A, B) and Fe_3_O_4_/SiO_2_ (C, D).

The morphology of the synthesized MNPs was characterized by STEM analysis. Different STEM images of bare Fe_3_O_4_ MNPs are shown in Figure 2A,B. Also, color STEM images (Figure 3A,C) and EDS spectra (Figure 3B,D) for unmodified (bare) and SiO_2_-modified Fe_3_O_4_ MNPs were given in Figure 3.

The STEM images of the Fe_3_O_4_ MNPs given in Figure 2 show the spherical structure of MNPs, with diameters ranging approximately between 8 and 12 nm. Similarly, Chang et al., who previously synthesized Fe_3_O_4_ with the same method, reported that the MNPs they obtained were spheres with diameters ranging between 10 and 15 nm.^6^ In Figure 2, an agglomeration indicating magnetic attraction among Fe_3_O_4_ particles can be seen.

**Figure 4:**
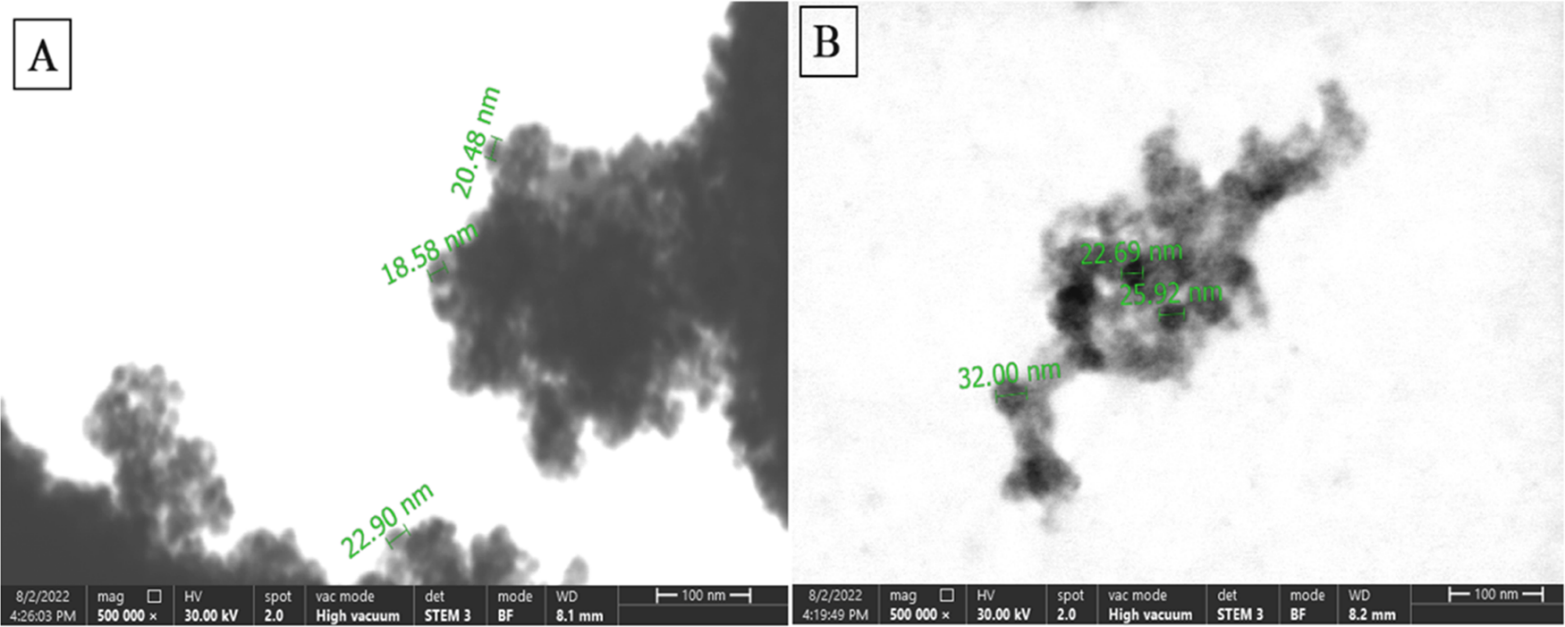
Different STEM images (A and B) taken for synthesized, UOx-immobilized Fe_3_O_4_ MNPs (UOx@ Fe_3_O_4_/SiO_2_/APTS/GLA).

The analysis results showed that the SiO_2_ modifications made by the TEOS reaction of MNPs were successfully carried out. Finally, UOx@Fe_3_O_4_/SiO_2_/APTS/GLA (MNPs after modification with SiO_2_, APTS, GLA, and attachment of UOx enzyme) is shown in Figure 4A,B. During the STEM analysis, a 500,000× magnification zoom level was applied.

Figure 4 reveals that while the average particle size for Fe_3_O_4_ nanoparticles was approximately 10 nm, it increased to about 18–25 nm as a result of enzyme immobilization after TEOS and APTS modifications. These findings can be interpreted as an indication of successful synthesis and modification for MNPs; enzyme (UOx) immobilization on MNPs was also confirmed by the surface film surrounding the MNPs (Figure 4).

On the other hand, the surface chemical compositions of the synthesized nanoparticles were analyzed using X-ray photoelectron spectrometry (XPS). XPS analyses were performed for (i) synthesized Fe_3_O_4_ MNPs (unmodified), (ii) MNPs modified by tetraethyl orthosilicate (TEOS, Fe_3_O_4_/SiO_2_), (iii) Fe_3_O_4_/SiO_2_ modified by the addition of 3-aminopropyltriethoxysilane (APTS, Fe_3_O_4_/SiO_2_/APTS), (iv) the same nanocomposite after modification with glutaraldehyde (GLA, symbolized as Fe_3_O_4_/SiO_2_/APTS/GLA), and lastly, (v) glucose oxidase-immobilized nanosensor in the final form (symbolized as Fe_3_O_4_/SiO_2_/APTS/GLA/GOx).

**Figure 5:**
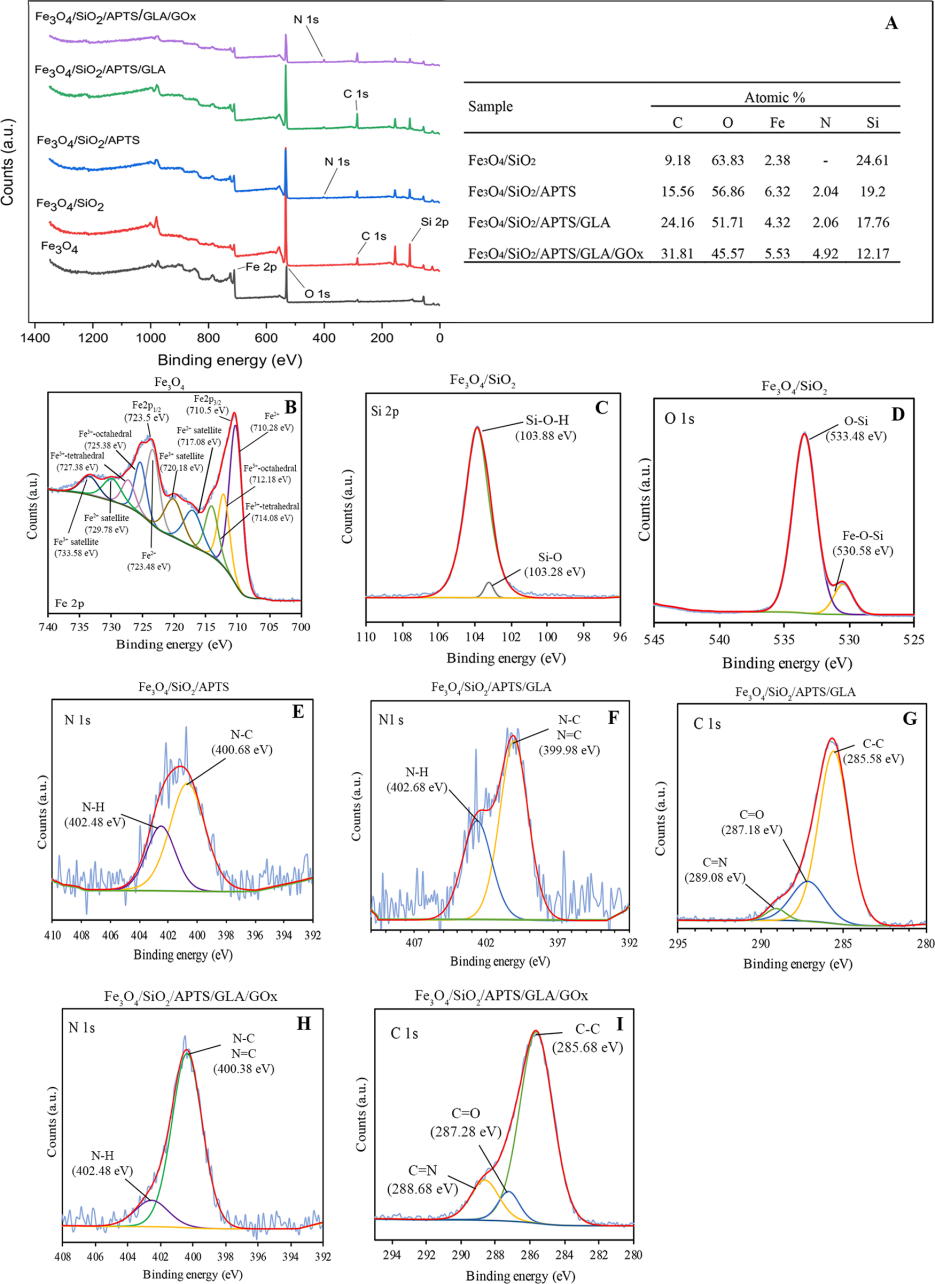
(A) XPS survey spectra and atomic contents of bare/modified Fe_3_O_4_ nanoparticles, after different modification steps. (B) Fe 2p spectrum of Fe_3_O_4_, (C) Si 2p spectrum of Fe_3_O_4_/SiO_2_, (D) O 1s spectrum of Fe_3_O_4_/SiO_2_, (E) N 1s spectrum of Fe_3_O_4_/SiO_2_/APTS, (F) N 1s spectrum of Fe_3_O_4_/SiO_2_/APTS/GLA, (G) C 1s spectrum of Fe_3_O_4_/SiO_2_/APTS/GLA, (H) N 1s spectrum of Fe_3_O_4_/SiO_2_/APTS/GLA/GOx, and (I) C 1s spectrum of Fe_3_O_4_/SiO_2_/APTS/GLA/GOx.

Figure 5A shows the XPS survey spectra and atomic contents of bare/modified Fe_3_O_4_ nanoparticles, after different modification steps. The dominant peaks seen in Fe_3_O_4_ are Fe 2p (723.48 and 710.48 eV), O 1s (529.98 eV), and C 1s (284.68 eV). The presence of Si 2p (103.88 eV), which is not seen in the first step in Fe_3_O_4_/SiO_2_, indicates that TEOS binds to the Fe_3_O_4_ surface. The nitrogen content of 2.04% N seen in Fe_3_O_4_/SiO_2_/APTS is due to the −NH_2_ group in the structure of APTS. The increase of the C 1s peak intensity in the survey spectrum of the Fe_3_O_4_/SiO_2_/APTS/GLA step also proves that glutaraldehyde binds to Fe_3_O_4_/SiO_2_. As a result of the immobilization of the enzyme in the last step, the intensity of the N 1s peak increased due to the −NH_2_ groups in the enzyme structure. Figure 5B–I shows the deconvolution of high-resolution peaks. In Figure 5B, the binding energies at 710.5 and 723.5 eV were attributed to Fe 2p_3/2_ and Fe 2p_1/2_, respectively. The binding energy peak at 710.3 eV belongs to the Fe^2+^ ions with the corresponding satellite at 717.08 eV. The peaks at 712.18 and 714.08 eV were attributed to the octahedral and tetrahedral Fe^3+^ ions, respectively. The satellite peak at 720.18 eV was associated with the Fe^3+^ ions. The peaks seen at 723.48, 725.38, 727.38, 729.78, and 733.58 eV in the 2p_1/2_ region can be attributed to Fe^2+^ ions, octahedral Fe^3+^ ions, tetrahedral Fe^3+^ ions, Fe^2+^ satellite peak, and Fe^3+^ satellite peak, respectively. Figure 5C shows the detailed XPS spectrum of Si 2p of Fe_3_O_4_/SiO_2_. Here, the peak seen at 103.88 eV was attributable to Si–OH, while the peak seen at 103.28 eV was associated with Si–O. When the O 1s spectrum for the same modification was examined, two peaks associated with Fe–O–Si and O–Si bonds were seen at 530.58 and 533.48 eV, respectively (Figure 5D). The peaks at 400.68 and 402.48 eV in the N 1s spectrum of Fe_3_O_4_/SiO_2_/APTS in Figure 5E were associated with N–C and N–H bonds, respectively. These bonds prove that the amination process with APTS takes place on the silica layer. N 1s and C 1s spectra of Fe_3_O_4_/SiO_2_/APTS/GLA are shown in Figure 5F,G, respectively. The peak attributed to N=C/N–C seen at 399.98 eV in the N 1s spectrum and the peaks associated with the C=O and C=N bonds, respectively, seen at 287.18 and 289.08 eV in the C 1s spectrum (Figure 5F) confirm the binding of GLA on the amine-functionalized nanoparticle. N 1s (Figure 5H) and C 1s (Figure 5I) spectra of Fe_3_O_4_/SiO_2_/APTS/GLA/GOx were examined to prove enzyme immobilization. It can be observed that the intensity of the N 1s peak increased as a result of the binding of −NH_2_ groups in the enzyme structure to the nanoparticle via GLA. Peaks in the spectrum at 400.38 and 402.48 eV can be attributed to N–C/N=C and N–H bonds. In Figure 5G, while the intensity of the peak of the C=O bond is higher than that of the C=N bond, the peak intensity of the C=N (288.68 eV) bond seen in the C 1s spectrum (Figure 5I) of the nanoparticle after enzyme immobilization is higher than that of the C=O (287.28) bond. This proves that the enzyme forms the −HC=N– bond by binding to glutaraldehyde through the −NH_2_ group.

### 3.2 Determination of the Corresponding Substrates of Oxidase-Attached MNPs

The experiments were performed as stated in Section 2.4. While the calibration graphs for Glu and ChCl (Figure S5) were drawn between the final concentrations of the substrates and CUPRAC absorbances (*A*), the graph of UA (Figure S6) was drawn between final concentrations and Δ*A* (i.e., the difference of the CUPRAC absorbances before and after UA treatment with UOx@MNP). The equations of linear calibration graphs, linear concentration ranges, and limit of detections (LODs) are collected in Table 1.

**Table 1:** Equations of the Obtained Calibration Graphs, Linear Concentration Ranges, and LOD Values for the Tested Substrates

tested substrate	equation of linear calibration graph (C: molar concentration)	linear range (μM)	LOD (μM)
Glu	*A* = 8.8 × 10^3^ C + 0.046 (*R*^2^ = 0.9968)	11.1–111.1	0.59
ChCl	*A* = 2.67 × 10^4^ C + 0.0281 (*R*^2^ = 0.9992)	2.78–44.4	0.20
UA	Δ*A* = 1.57 × 10^4^ C + 0.043 (*R*^2^ = 0.9975)	5.43–65.22	0.34

**Figure 6:**
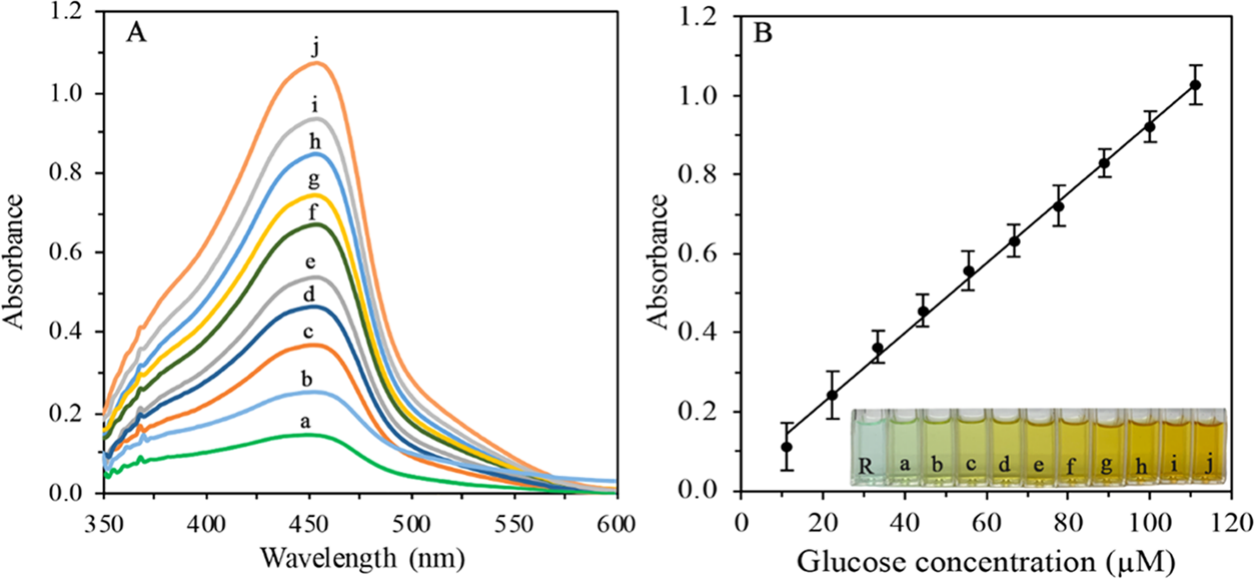
Spectra obtained by the measurement of CUPRAC absorbance of H_2_O_2_ released by the enzymatic reaction between varying concentrations of Glu and GOx@MNPs (A) and the related calibration graph (B) (The final concentrations of Glu as μM tested a: 11.1, b: 22.2, c: 33.3, d: 44.4, e: 55.6, f: 66.7, g: 77.8, h: 88.9, i: 100.0, and j: 111.1. The increasing color intensity is also shown at the bottom of Figure 6B).

The obtained spectra and the corresponding calibration graph between the final concentrations of Glu and CUPRAC absorbance are exemplified in Figure 6, whereas other similar calibration graphs were given in Section S.II.1.

It should be emphasized that the mentioned calibration graphs had very good linearity (*R*^2^ values were between 0.9968 and 0.9992) due to the very clear stoichiometry of the reaction. Although the linear range could cover higher concentrations than the tested ones, the upper limit of absorbance was not let to exceed 1.0 au to prevent possible deviations from Beer’s law.

In addition to the calibration equations depicted in Table 1, the findings of other literature reports with a similar mechanism used for the determination of Glu, ChCl, and UA, together with their linear ranges and LOD values, are displayed in Table S1.

### 3.3 Determination of UA in the Presence of Different AOx Mixtures by the Proposed UOx@MNPs Method

To test selective determination of UA in the presence of other AOx compounds, a series of binary mixtures were prepared, and the experiments were conducted as described in Section 2.6. Here, the CUPRAC absorbance values obtained after UOx@MNPs treatment were expressed as *A*_UOx-CUPRAC_, and the absorbance values without UOx treatment were shown as *A*_CUPRAC_. The difference between the two values, i.e., (*A*_CUPRAC_ – *A*_UOx-CUPRAC_) symbolized as Δ*A*, was used to calculate UA concentration. For different AOx mixtures, *A*_CUPRAC_, *A*_UOx-CUPRAC_, Δ*A*, the concentration of UA added to the mixture (theoretical) and that calculated by Δ*A* values (experimental) were shown with relative errors in Table S2.

When Table S2 was examined, it can be seen that the relative error values calculated for theoretical and experimental UA concentrations were between 0.18 and 4.87%. These relatively low error values showed that the method can be used for UA determination in the presence of other phenolic or thiol-type AOx compounds.

As described in Section S.I.4, UA determination in the presence of some serum AOxs and experimental UA concentrations were calculated as stated above. The obtained results were collected in Table S3.

The calculated percentage error values for UA in the presence of serum antioxidants were between 2.40 and −4.35%, confirming that the proposed method can be applied to UA determination in serum samples and that UOx@MNPs is perfectly selective for UA.

### 3.4 Chromatographic UA Determination

The HPLC method was applied as described in Section S.I.5. According to this, the obtained standard calibration graph for standard UA using HPLC is shown in Figure S7. The equation of the plot was calculated as *A* = 2.0 × 10^10^*C* – 9.9 × 10^3^, (C: molar concentration and *A*: peak area) with a determination coefficient *R*^2^ = 0.9997.

### 3.5 Application of the Proposed Method to the Real Samples

#### 3.5.1 Application of the Proposed GOx@MNPs Method to Real Samples

As it was stated in Section S.I.6.1, the applicability of the proposed method to samples with complex matrices was demonstrated in an FBS sample containing Glu. Standard Glu was added to FBS and recovery percentages were calculated. Glu determination in FBS was performed five times, and results were given after necessary statistical calculations. The results were given as follows: *x̅* = (*t*_0.95_ × s/√*N*); *N* = 5 (*x̅* = mean, *s* = standard deviation). According to this, the Glu concentration was determined as 141.0 ± 4.2 mg dL^–1^, while the value declared by the manufacturer was 139 mg dL^–1^.

As can be seen, the amount of glucose determined by the proposed method was found to be very close to that specified by the manufacturer in FBS. As stated earlier, standard addition was applied according to the obtained results, and the recovery values were 101.4 and 101.2%. This showed us that the proposed method can be applied conveniently to Glu determination in samples such as biological and food samples. The standard addition test results are given in Table S4.

#### 3.5.2 Application of the Proposed ChOx@MNPs Method to Real Samples

The proposed method was used to determine Ch in a commercially available infant formula obtained from a local market in Istanbul. The method was applied as described in Section S.I.6.2. According to the obtained results for five replicate samples, the Ch content of the infant formula was calculated as 16.1 ± 0.5 mg/100 mL. This value was compatible with that declared by the manufacturer as 15 mg/100 mL, meaning that the proposed method can be applied to complex samples containing ChCl.

#### 3.5.3 Determination of UA-Free TAC in FBS

To determine UA-free TAC, the proposed method was applied directly to FBS as the real sample and to UA-spiked FBS. The total TAC was calculated in AA equivalent units by applying the CUPRAC method to FBS and UA-added samples. Also, for UA-free TAC, the CUPRAC method was applied after the samples were treated with UOx@MNPs. The obtained results are tabulated in Table S5.

The HPLC method described earlier was applied to FBS directly and after UOx@MNPs treatment, and the obtained chromatogram is shown in Figure S8.

The obtained chromatograms revealed that UA was completely decomposed after UOx@MNPs treatment. An HPLC peak with a retention time of approximately 8.5 min was observed in the chromatogram of the sample not treated with UOx@MNPs. This peak belonging to UA was no longer seen in the sample treated with UOx@MNPs.

In addition, the method of standard addition was applied; 10.9 and 21.7 μM standard UA was added into 0.5 mL of UA separately, and the obtained chromatograms are shown in Figure S9. The recovery values for spiked FBS samples were calculated by the proposed UOx@MNPs method and the standard HPLC method. The recoveries were calculated as 98.07 and 98.43% for the proposed UOx@MNPs method and 102.1 and 103.5% for the HPLC method. These results confirm that UA concentrations could be accurately calculated by the proposed UOx@MNPs method in accordance with those of the standard HPLC method (Table S6).

### 3.6 Investigation of Enzyme Kinetics

The Michaelis constant (*K*_m_) is the substrate concentration at which the reaction rate is half of the maximum value and is considered to be a measure of the substrate’s affinity to the enzyme. A low *K*_m_ value indicates a high affinity to the substrate and represents that the reaction rate reaches *V*_max_ more rapidly. *V*_max_ is the maximum velocity of an enzymatically catalyzed reaction when the enzyme is saturated with its substrate. Since the maximum velocity is described to be directly proportional to the enzyme concentration, it can therefore be used to estimate enzyme concentration.

The kinetic parameters of the enzyme (*K*_m_ and *V*_max_) generally undergo changes after immobilization, indicating a change in affinity for the substrate. These changes may occur due to various factors such as structural changes resulting from bonding to the support, steric hindrance, and diffusion effects. These factors cause *K*_m_ to decrease or increase.

Enzyme kinetics is commonly calculated based on the Michaelis–Menten kinetic equation. Michaelis–Menten kinetics is one of the simplest and best models of enzyme kinetics. In the Michaelis–Menten kinetic equation, the reaction rate (*V*) is expressed in terms of the substrate ([S]) concentration:
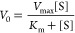
When the equation is inverted, the Lineweaver–Burk equation is obtained, which is a linearized form of the original equation as *V*_0_^–1^ versus [S]^−1^.
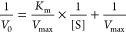


In the kinetic study for free and immobilized oxidase enzymes tested, the procedures given in Section 2.9 were applied. The Michaelis–Menten graphs obtained by plotting V_0_ against different concentrations of the substrate [S] using free and immobilized UOx enzymes are given in Figure S10, and the linearized Lineweaver–Burk graphs of free and immobilized enzymes are given in Figure S11.

Since (*K*_m_/*V*_max_) is the slope and (1/*V*_max_) is the intercept of the linearized graph (Figure S12), *K*_m_ and *V*_max_ values were calculated as 174 μM and 31.06 μM min^–1^, respectively, for the free UOx enzyme. On the other hand, for immobilized UOx, *K*_m_ was calculated as 79 μM and *V*_max_ was 16.89 μM min^–1^. According to the obtained results, the *K*_m_ value of the immobilized enzyme was lower than that of the free enzyme. A low *K*_m_ value indicates that it has a greater affinity for the substrate and saturates more quickly. Compared to the free enzyme, the *V*_max_ values of the immobilized enzyme were found to be 45.6% smaller. This can be explained by the decreased flexibility of the immobilized enzyme on a solid surface, thereby reducing the accessibility of active surface sites.

The same approach was adapted to two other enzymes to calculate the kinetic parameters by means of the procedure explained in Section S.I.7.1. For this purpose, the formation rates of H_2_O_2_ generated by enzyme–substrate reactions were separately measured as described earlier. The calculated kinetic parameters for the tested three oxidases are depicted in Table 2.

**Table 2:** Linear Equations of Lineweaver–Burk Graphs, Michaelis Constant (*K*_m_), and Maximum Velocity of an Enzymatically Catalyzed Reaction (*V*_max_) Values of the Tested Enzymes

enzyme		linear equations of Lineweaver–Burk graphs	*K*_m_ (mM)	*V*_max_(μM min^–1^)
UOx	free	1/*V* = 0.0056 × 1/[S] + 0.0322, (*R*^2^ = 0.9949)	0.174	31.06
	immobilized	1/*V* = 0.0047 × 1/[S] + 0.0592, (*R*^2^ = 0.9911)	0.079	16.89
GOx	free	1/*V* = 0.3359 × 1/[S] + 0.0277, (*R*^2^ = 0.9988)	12.1	36.1
	immobilized	1/*V* = 0.5896 × 1/[S] + 0.0486, (*R*^2^ = 0.9948)	12.1	20.6
ChOx	free	1/*V* = 0.0074 × 1/[S] + 0.0302, (*R*^2^ = 0.9924)	0.28	33.1
	immobilized	1/*V* = 0.0099 × 1/[S] + 0.0351, (*R*^2^ = 0.9969)	0.25	28.5

As can be seen from Table 2, the *K*_m_ values for the immobilized and free GOx enzymes were the same, and for ChOx, these values were very close. This proves that there was no (or only a little) change in the affinity of the enzymes to their substrates after immobilization.

On the other hand, when the *V*_max_ values were examined for GOx and ChOx, the *V*_max_ of the immobilized enzymes decreased by 43 and 14%, respectively, when compared to those of the free enzymes. Actually, this is an expected situation, since immobilization limits the movement of the enzyme attached to a solid surface.

### 3.7 Investigation of the Stability and Reusability of MNPs-Attached Enzymes

As stated in the literature, nanoparticles having enzymatic properties (nanozymes) were shown as alternatives for natural enzymes. Although nanozymes are cheaper alternatives for natural enzymes, they are not comparable in selectivity with natural enzymes. However, the usage of the same enzyme several times can reduce expenses drastically. Considering this point, the stability, reusability, intraday repeatability, and interday reproducibility of the enzyme immobilized on MNPs were tested as described in Section 2.10. The experiments were conducted for all three immobilized oxidase enzymes for two different substrate concentrations. The obtained results showed that UOx-, GOx-, and ChOx-attached MNPs could be used at least five times with a relative standard deviation (RSD %) under 10%. On the other hand, for diluted analytes, RSD % values were lower and the reusability could be more than five times (the obtained results are collected in Table S7).

To examine the stability of the immobilized enzymes stored at 4 °C, the method was applied using substrates at a constant concentration at different times. After 60 days, there was 3, 4, and 6% decrease in absorbance (or Δ*A* for UA) values for UA, Glu, and ChCl, respectively. The obtained results are given in Figure S12.

As a result of intraday reproducibility and interday reproducibility experiments, RSD % values were found as 3.12, 4.61, and 3.84 for UA, Glu, and ChCl, respectively, and the intraday results in the same order were 3.61, 5.32 and 4.28, respectively.

## 4 Conclusions

In the presented study, three different natural oxidase enzymes, namely, GOx, ChOx, and UOx, were attached to MNPs separately, and an enzymatic colorimetric method was developed to determine the related enzyme substrates (Glu, ChCl, and UA, respectively). All three substances analyzed are highly important for biology, human health, and food industry. Biological enzymes have unique selectivity for their substrates, and this property allows the determination of the substrate analytes in complex matrices. Although certain nanoparticles with enzyme-like activities (also known as nanozymes) were presented as economical alternatives for natural enzymes, nanozymes cannot effectively compete with biological enzymes in terms of their lower substrate selectivity and turnover ratios. Generally, immobilization of enzymes on a solid support makes them reusable and reduces the cost. In this study, GOx, ChOx, and UOx were attached to MNPs covalently; thus, the loss of enzymes upon repetitive use was minimized. In general terms, the developed method was based on the determination of enzymatically generated H_2_O_2_ by the CUPRAC colorimetric method. Here, the light blue-colored CUPRAC reagent, Cu(II)–Nc complex, is reduced to the stable yellow/orange-colored Cu(I)–Nc chelate showing strong charge-transfer absorption. The reaction mechanism involves a simple electron transfer with a clear stoichiometry because Cu(II)–Nc is one of the rare oxidants capable of converting hydrogen peroxide to molecular oxygen without requiring a H_2_O_2_ activator, and this 2-e oxidation ensures high molar absorptivity for H_2_O_2_ indirectly via the reduced cuprous–neocuproine chelate. On the other hand, the majority of literature studies for enzymatic colorimetric determinations via H_2_O_2_ need oxidation of a chromogenic peroxidase substrate such as TMB, ABTS, and 4-AAP in the presence of a catalyst. In addition, sometimes this catalyst is another enzyme like HRP, bringing the risk of enzyme inhibition to the analysis from natural sources. These kinds of reactions, mostly utilizing nanozymes, may also operate nonstoichiometrically most of the time because H_2_O_2_ may not only be degraded into a variety of reactive oxygen species (ROS) during the catalytic determination, oxidizing TMB-like peroxidase substrates, but in addition, the converted products may enter redox cycling with H_2_O_2_. However, a simple redox reaction (used in this work) depends on single-product formation (i.e., Cu(I)–Nc) and is much less complicated and more stoichiometric. Beer’s law of optical densities of solutions is perfectly obeyed by single-product formation. At the same time, since the CUPRAC method was originally developed for TAC determination, another important parameter, UA-free TAC, could also be determined using the same method. In addition, after immobilization, the possibility of reduction of enzyme affinity by immobilization could be investigated by kinetic experiments. Owing to these kinetic studies, the affinities of free and immobilized enzymes were compared. So, it could be concluded that there was no (or only a little) change in the affinity of the enzymes to their substrates after immobilization. However, the *V*_max_ values determined for immobilized enzymes showed a decrease compared to those calculated for free enzymes, possibly due to the accessibility of active sites. Finally, the developed method was applied for Glu, Ch, and UA determination in real samples having a complex matrix. The results of this study may pave the way to further biochemical studies investigating oxidase enzyme–substrate reactions aided by robust analysis.

## Supporting Information Available

Preparations of solutions and reagents; linear calibration graphs of UA and ChCl; determination of UA in the AOx mixture; and application of the proposed method to selected real samples (PDF)
