# Supplementary material for: Colorimetric Nanobiosensor Design for Determining Oxidase Enzyme Substrates in Food and Biological Samples
Source: ACS Omega. 2022 Nov 23;7(48):44372–82. doi: 10.1021/acsomega.2c06053 (PMC9730783; doi:10.1021/acsomega.2c06053)
Supplement: Supplementary file 1 — ao2c06053_si_001.pdf [file ao2c06053_si_001.pdf]

SUPPLEMENTARY MATERIAL

Colorimetric Nanobiosensor Design for Determining Oxidase Enzyme Substrates in Food and Biological Samples

Aslı Neslihan Avan[a,b], Sema Demirci-Çekiç[a,*], Reşat Apak[a,c,]**

[a] Department of Chemistry, Faculty of Engineering, Istanbul University-Cerrahpasa, 34320 Avcilar, Istanbul, Turkiye
[b] Department of Chemistry, Institute of Graduate Studies, Istanbul University-Cerrahpasa, Turkiye
[c] Turkish Academy of Sciences (TUBA), Vedat Dalokay St. No. 112, 06670 Cankaya, Ankara, Turkiye

* Correspondence: sema@iuc.edu.tr; Tel: +90-212-4737028

Table of Contents of Supplementary Material

S.I.1. Preparations of solutions and reagents used in the experiments

S.I.2. Examination of the effects of experimental conditions on the immobilized enzymes

S.I.3. Preparation of calibration graphs

S.I.3.1. Calibration graphs for Glu and ChCl

S.I.3.2. Calibration graph for UA

S.I.4. Determination of UA in the AOx mixture

S.I.5. Chromatographic UA determination

S.I.6. Application of the proposed method to selected real samples

S.I.6.1. Glu determination in FBS by the proposed GOx@MNPs method

S.I.6.2. Ch determination in commercially available infant formula by using the proposed ChOx@MNPs method

S.I.6.3. Determination of UA and UA-free TAC in FBS by the proposed UOx@MNPs method

S.I.6.4. Determination of UA in FBS by HPLC method

S.I.7. Investigation of enzyme kinetics

S.I.7.1. Determination of enzyme kinetics for GOx and ChOx

S.II.1. The effects of pH, temperature and different solvents on the immobilized enzymes

S.II.2. Calibration graphs drawn for ChCl and UA

S.II.2.1. Linear calibration graph of ChCl obtained by the proposed ChOx@MNPs method

S.II.2.2. Linear calibration graph of UA obtained by the proposed UOx@MNPs method

S.II.3. Linear range and LOD values obtained by the proposed method and given in the selected enzymatic colorimetric studies for the tested substrates

S.II.4. Determination of UA in the AOx mixture

S.II.5. Chromatographic UA determination
S.II.6. Application of the proposed GOx@MNPs method to real samples
S.II.7. Determination of TAC and UA-free TAC in FBS by the proposed UOx@MNPs method
S.II.8. Investigation of enzyme kinetics
S.II.9. Investigation of stability and reusability of MNPs-attached Enzymes

S.I.1. Preparation of solutions and reagents used in the experiments

CUPRAC method reagents ($CuCl_2.2H_2O$, neocuproine (Nc), pH 7.0 ammonium acetate (NH_4Ac) buffer) were prepared as described by Apak et al. previously, [1] and pH 7.0 urea buffer was prepared as stated by Demirci-Çekiç et. al.[2]

Phosphate buffer solution (PBS): To prepare phosphate buffer solution at different pH values (such as pH 7.0 and 8.5), suitable volumes of NaH_2PO_4 and Na_2HPO_4 solutions at 0.1 M concentrations were mixed.

Antioxidant (AOx) Solutions: Stock solutions of catechin (CAT), quercetin (QR), trolox (TR), ferulic acid (FA), caffeic acid (CFA) and gallic acid (GA) at 1.0×10^{-2} M concentration were prepared with ethanol. To obtain the working solutions of antioxidants, stock solutions were diluted with ethanol at different ratios just before analysis. To prepare uric acid (UA) and bilirubin stock solutions (5.0×10^{-3} M and 1.0×10^{-3} M respectively), appropriate amounts of solids were weighed and dissolved in 0.5 mL of 1.0 M NaOH, then the volumes were completed to 25 mL with distilled water (to prepare working solutions, stock solutions were diluted by distilled water at necessary ratios). Glutathione (GSH), N-acetyl-L-cysteine (NAC) and ascorbic acid (AA) solutions were prepared in distilled water. To prepare 1.0×10^{-2} M cysteine (CYS) solution, an appropriate amount of CYS was dissolved in 0.5 mL of 1.0 M HCl solution, then diluted to 25 mL with distilled water.

Catalase (CAT) solution: For the preparation of catalase solution at a concentration of 1 mg/mL, the appropriate weight taken from 2000-5000 U/mg of catalase was dissolved with 0.1 M pH 7.0 phosphate buffer and diluted at 1:10 ratio with pH 7.0 phosphate buffer just before use.

Choline chloride (ChCl) solution: To prepare the stock solution at a concentration of 1.0×10^{-2} M, a weight of 0.0140 g was dissolved with distilled water and diluted to 10 mL.

S.I.2. Examination of the effects of experimental conditions on the immobilized enzymes

As is known, although biological enzymes have unique selectivity against their substrates, they are not resistant to harsh experimental conditions. For this purpose, the effects of temperature, pH and organic solvents on the immobilized enzymes were investigated. During the experiments, the proposed methods for UA, Glu and ChCl determinations in the presence of oxidase enzymes (uricase, glucose oxidase and choline oxidase, respectively) immobilized on Fe_3O_4-magnetic nanoparticles (MNPs) (named as: UOx@MNPs, GOx@MNPs and ChOx@MNPs,) were applied as stated in the manuscript.

The experimental conditions used can be summarized as follows: Firstly, the method was carried out at 25, 37, 50 and 60 °C for 54.3 µM UA, 88.9 µM Glu and 33.3 µM ChCl. For pH experiments, the method was applied in the presence of different buffer solutions between pH 4.0 and 10.0 (for pH 4.0 – 6.0, 0.2 M acetic acid/sodium acetate, for pH 7.0, 1.0 M NH_4Ac, and for pH 8.0 – 10.0, 0.2 M NH_3/NH_4Cl buffer solutions were used). 65.2 µM UA, 88.9 µM glucose and 33.3 µM ChCl were used in the experiments. Finally, the tested organic solvents were ethanol, methanol, acetone and acetonitrile. The method was applied by adding 0.5 mL of the solvents to 54.3 µM UA, 88.9 µM Glu, 33.3 µM ChCl solutions.

S.I.3. The preparation of the calibration graphs

S.I.3.1. Calibration graphs for Glu and ChCl

To construct the Glu and ChCl calibration graphs, the procedure described in Section 2.4. was applied for the volumes taken from the related solutions separately. For this purpose, to obtain Glu standard calibration graph, different volumes varying between 50 µL and 0.5 mL were taken from the standard Glu solution at 1.0×10^{-3} M concentration.

On the other hand, for drawing the ChCl calibration graph, different volumes between 25 µL and 0.4 mL were taken from a standard ChCl solution at a concentration of 5.0×10^{-4} M. After measurement of CUPRAC absorbances, the calibration graphs were drawn between final concentrations of the standard substrate solutions and measured absorbances. Then the equation of the calibration curve was calculated with the determination coefficient.

S.I.3.2. Calibration graph for UA

For this purpose, different volumes varying between 50 μL and 0.6 mL were taken from the UA solution at a concentration of 5.0×10^{-4} M, and the procedure described in Section 2.4.3. was applied in the presence and absence of UOx@MNPs.

S.I.4. Determination of UA in the AOx mixture

To prepare a synthetic AOx mixture solution, different volumes taken from 5.0×10^{-4} M UA; 1.0×10^{-3} M CFA, QR, GA, CAT, FA, NAC, CYS, GSH and 1.0×10^{-2} M TR were mixed. Firstly, standard CUPRAC procedure was applied to the mixture,[1] then the method described in Section 2.4.3 was applied to another identical AOx mixture. The UA concentration was calculated by using the difference between the two absorbance values measured.

Additionally, another mixture, containing UA in the presence of certain plasma AOxs, namely Bil, AA and GSH were also investigated. For this purpose, 0.1 mL of 5×10^{-4} M UA was mixed with 0.1 mL of 1.0×10^{-3} M GSH, 0.2 mL of 4.0×10^{-4} M AA and 0.2 mL of $1,0\times10^{-4}$ M Bil separately. Here, the CUPRAC method was applied directly to the mixtures, and then the mixtures were treated with UOx@MNPs, followed by re-application of the CUPRAC method.

S.I.5. Chromatographic UA determination

To compared the results, get by the proposed method a reverse phase HPLC method was used as the standard verification method. For testing the amount of UA in the AOx mixtures, the method described earlier by George *et al.*[3] was applied with a few modifications. A C18 (250 mm × 4.6 mm × 5 μm) column was used as the stationary phase and the mobile phase was 10 mM KH_2PO_4 at pH 4.7. The column temperature was kept constant at 25ºC during the 15-minute isocratic elution at a flow rate of 0.8 mL min^{-1}. Analyses were performed using a photodiode array (PDA) detector within the wavelength range of 200-800 nm, and 280 nm was used as the detection wavelength. Finally, the injection volume was 20 μL.

To obtain UA calibration graph, UA standards at different concentrations between 1.0×10^{-6} and 1.0×10^{-4} M were injected into the HPLC system at the conditions stated above. Then the calibration graph for HPLC method between UA concentration and peak areas was drawn.

S.I.6. Application of the proposed method to selected real samples

S.I.6.1. Glu determination in FBS by the proposed GOx@MNPs method

For this purpose, an aliquot of 2 mL was taken from FBS solution and the protein fraction was precipitated by the addition of 1 mL of 20% TCA. After separation of precipitated proteins by centrifugation, a volume of 2.3 mL was taken from the supernatant and neutralized with the addition of 3 M NaOH. The final volume was completed to 2.5 mL and the method was applied as stated earlier for 50 µL of the sample. Then 0.1 and 0.15 mL of 1.0×10^{-3} M Glu were added to the same sample for determination in spiked samples.

S.I.6.2. Ch determination in commercially available infant formula by using the proposed ChOx@MNPs method

With the developed method, Ch determination was carried out in commercially available infant formula used for 6-9 months old babies. Since choline may be in ester form in some food samples, it was aimed to release Ch in the sample by hydrolysis. For this purpose, 5 g of sample was taken and after addition of 30 mL of 1 M HCl, the mixture was incubated in a 70ºC water bath for 3 h with continuous stirring. At the end of the period, the sample was centrifuged and 10 mL of the supernatant was neutralized with 3 M NaOH. Finally, sample volume was completed to 15 mL with distilled water. The determination was made in final solution by the developed method and by adding standard ChCl.[4]

S.I.6.3. Determination of UA and UA-free TAC in FBS by the proposed UOx@MNPs method

For the determination of UA with the developed method, the FBS sample was used undiluted; 0.5 mL was taken, and the method was applied as described in Section 2.4.3.

The CUPRAC method was applied to the serum sample before and after it was treated with UOx@MNPs. The UA concentration in FBS was calculated from the difference in absorbance.

S.I.6.4. Determination of UA in the FBS by HPLC method

For this purpose, FBS was directly injected to the HPLC system first of all and then re-injected after treatment with UOx@MNPs.

In addition, standard addition experiments were performed. For this purpose, 10.9 µM and 21.7 µM UA standards were added to 0.5 mL of FBS. The spiked samples were analyzed by HPLC and the proposed method.

S.I.7. Investigation of enzyme kinetics

S.I.7.1. Determination of enzyme kinetics for GOx and ChOx

To investigate enzyme kinetics, Michaelis constant (K_m) and maximum velocity of an enzymatically catalyzed reaction (V_{max}) values were calculated for free and immobilized enzymes. In this part, the frequently used spectrophotometric methods to investigate enzyme kinetics were preferred.

For this purpose, H_2O_2 generated by the reaction between related enzymes (GOx and ChOx) and their substrates (glucose (Glu) and choline chloride (ChCl) respectively) a common spectrophotometric method was used. According to the method proposed by Trinder[5], a red quinone-imine dye was formed at the end of the reaction given below. The absorbance of the final product was measured at 506 nm.

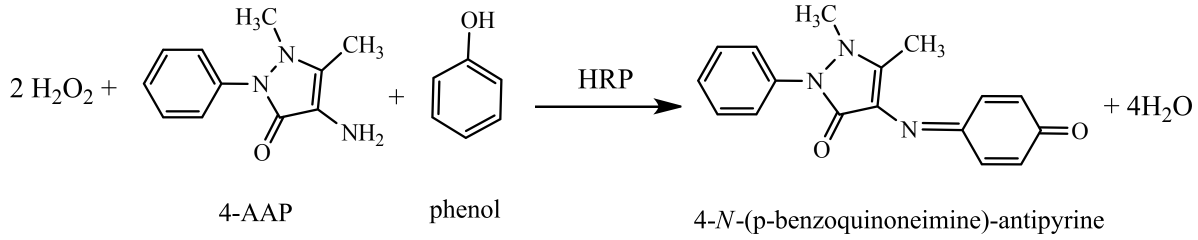

Figure S1. The formation of red colored 4-*N*-(*p*-benzoquinoneimine)-antipyrine through the reaction between enzymatically generated H_2O_2, 4-aminoantipyrine (4-AAP) and phenol in the presence of horseradish peroxidase (HRP)

The related procedure can be summarized as follows:

For GOx: 0.5 mL of 0.25 M phosphate buffer solution (PBS) at pH 7.4 + 0.5 mL of 1.2×10^{-3} M 4-AAP + 0.5 mL of 2.0×10^{-3} M phenol + 100 µL HRP (37.5 U mL^{-1}) + x mL 0.1 M Glu + (0.85-x) mL H_2O + 50 µL GOx (0.2 mg mL^{-1}) were mixed and incubated for 10 min at room temperature, and the absorbance due to generated quinone-imine was read at 506 nm.

For ChOx, the same method was applied with a few differences. Instead of 0.5 mL of PBS, 0.2 M NH_3/NH_4Cl buffer solution (at pH 9.0) at the same volume was used. Additionally, Glu was replaced with ChCl and similarly ChOx (4.8 U mL^{-1}) was used instead of GOx.

S.II.1. The effects of pH, temperature and different solvents on the immobilized enzymes

The results of the temperature dependency experiment were given in Figure S2.

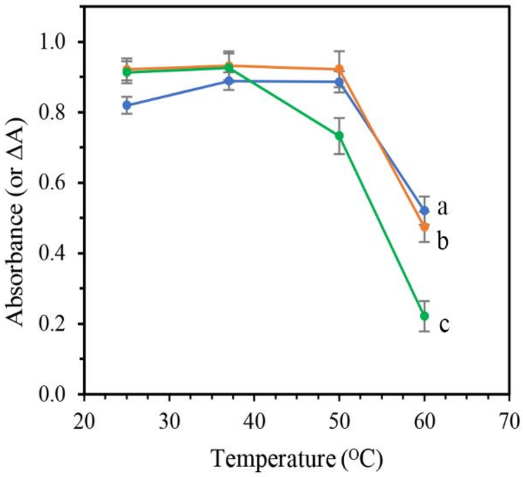

Figure S2. Change in the CUPRAC absorbance (ΔA for UA) of a) UA in the presence of UOx@MNPs, b) Glucose in the presence of GOx@MNPs and c) ChCl in the presence of ChOx@MNPs at different temperatures.

When the graph was examined, it was observed that the activity of immobilized ChOx started to decrease after 37 °C. For temperatures higher than 50 °C, a significant decrease in the activity of all three immobilized enzymes (UOx, GOx and ChOx) was observed.

The results of the pH dependency experiment were given in the Figure S3.

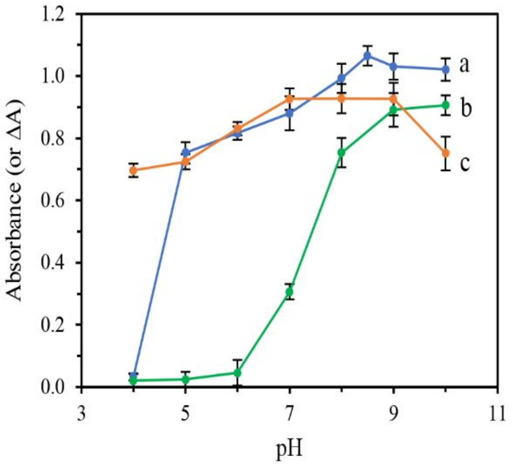

Figure S3. Change in the CUPRAC absorbance (ΔA for UA) of a) UA in the presence of UOx@MNPs, b) ChCl in the presence of ChOx@MNPs and c) Glucose in the presence of GOx@MNPs at different temperatures.

It has been observed that the most effective pH ranges of immobilized enzymes were between pH 8.0-10.0 for ChOx, pH 7.0-9.0 for GOx, and pH 8.0-10.0 for UOx.

The results obtained as 'solvent free' and in the presence of different organic solvents were given in Figure S4 as a bar graph.

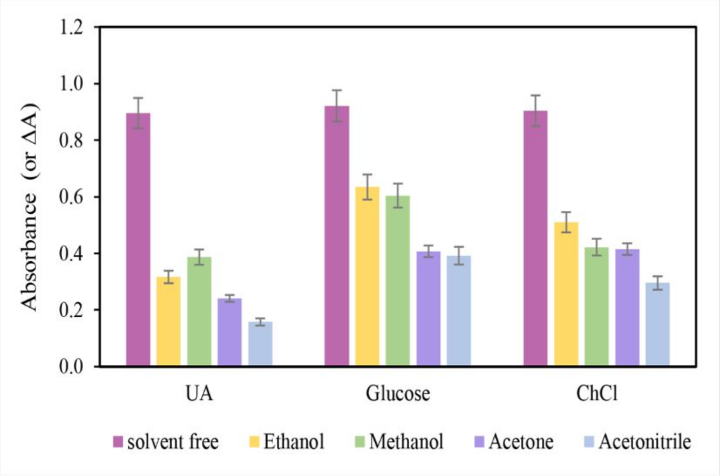

Figure S4. Change in CUPRAC absorbance of UA, ChCl and Glu as 'solvent free' and in the presence of acetone, ethanol, methanol.

It was observed that the activity of all three immobilized enzymes decreased in the presence of organic solvents.

S.II.2. Calibration graphs drawn for ChCl and UA

S.II.2.1 Linear calibration graph of ChCl obtained by the proposed ChOx@MNPs method

To prepare a calibration graph for ChCl, different volumes of ChCl taken from a standard ChCl solution at a concentration of 5.0×10^{-4} M were treated with ChOx@MNPs, and H_2O_2 generated as a result of ChCl reaction with ChOx was determined by the spectrophotometric CUPRAC method. The calibration graph between the final concentration of ChCl and resulting CUPRAC absorbance was given below

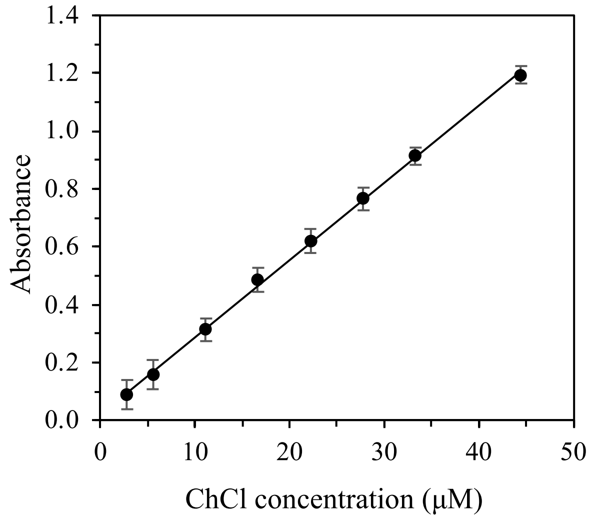

Figure S5. ChCl calibration graph obtained by the ChOx-attached MNP method, drawn as CUPRAC absorbance *versus* final concentration of ChCl.

S.II.2.2. Linear calibration graph of UA obtained by the proposed UOx@MNPs method

The experiments were conducted as stated in Section S.I.2.2. For different volumes taken from the UA standard solution at a concentration of 5.0×10^{-4} M, classical CUPRAC method[1] was applied directly; for the same volume of the standard solution, the procedure described in Section 2.4.3 was applied, and CUPRAC method was applied to UOx@MNPs-treated UA sample. The absorbance differences between UOx@MNPs treated and untreated samples were calculated and plotted against the final concentration of UA. The obtained graph was shown in **Figure S6.**

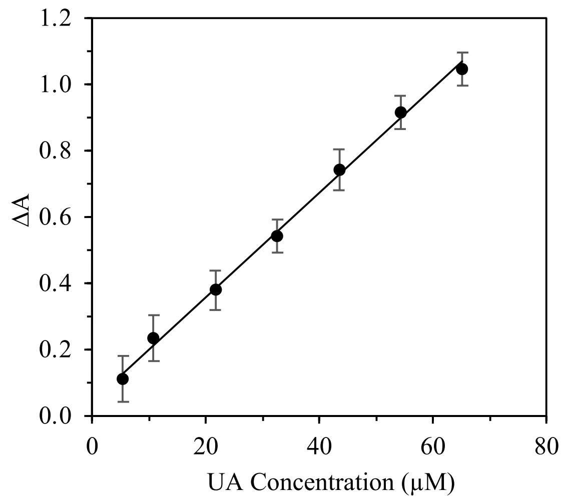

Figure S6. UA calibration graph obtained using the UOx-attached MNP method, for which the absorbance differences between UOx@MNPs treated and untreated UA samples were plotted against the final concentrations of UA.

S.II.3. Linear range and LOD values obtained by the proposed method and by the selected enzymatic colorimetric literature studies for the tested substrates

Table S1. Analytical figures of merit of the proposed method for determining Glu, ChCl and UA, compared to those of similar literature methods

Substrate tested	Tehnique used	Mechanism	Linear range (µM)	LOD (µM)	ref
**Glucose**	Colorimetry	Fe_3O_4 MNPs catalyzed reaction between ABTS and enzymatically generated H_2O_2	5-100	3.0	[6]
	Colorimetry	Peroxidase like activity of $ZnFe_2O_4$ MNPs. enzymatically generated H_2O_2 was reacted with $ZnFe_2O_4$ MNPs to colorize TMB	1.25-18.75	0.3	[7]
	Colorimetry (smart phone application)	GOx-$Cu_3(PO_4)_2{\cdot}3H_2O$ hybrid microflowers produced a colored product by the reaction with Glu and TMB	1.0-1000	0.3	[8]
	CUPRAC Colorimetry	Determination of enzymatically produced H_2O_2 by the reaction between GOx immobilized MNPs and Glu by CUPRAC method	11.1 - 111.1	0.59	This study

**Choline**	Colorimetry	Ch and molecular oxygen converted colorless ABTS into green $ABTS^{\bullet +}$ in the presence of DNAzyme	0.1–25	0.022	[9]
	CUPRAC Colorimetry	Determination of enzymatically produced H_2O_2 by the reaction between ChOx immobilized MNPs and ChCl by CUPRAC method	2.78 – 44.4	0.2	This study
**Uric acid**	Colorimetry	Determination of H_2O_2, produced by UOx and UA, was determined by using HRP	50-250	8.0 mg/0.1 mL	[10]
	Colorimetry	The reaction between UOx immobilized onto a vial and a color reagent consisting of 4-aminophenazone, *p*-hydroxybenzoic and HRP	10-1200	10	[11]
	Colorimetry	The peroxidase like activity of UOx embedded into a copper nanozyme in the presence of 4-aminoantipyrine as coloring agent	1–50	0.6	[12]
	Colorimetry	The peroxidase like activity of ultra small CuS NPs towards TMB in the presence of enzymatically generated H_2O_2	1-100	0.1	[13]
	Colorimetry	The peroxidase mimetic activity of BSA-stabilized Au nanoclusters (Au NCs); enzymatically produced H_2O_2 was reacted with TMB in the presence of BSA-Au NCs	2.0–200	0.36	[14]
	Fluorimetry	H_2O_2 produced by enzymatic reaction caused quenching of fluorescence of CdTe NPs capped with different thiol containing ligands	0.22-6	0.1	[15]
	CUPRAC Colorimetry	Determination of enzymatically produced H_2O_2 by the reaction between UOx immobilized MNPs and UA by CUPRAC method	5.43-65.22	0.34	This work

S.II.4. Determination of UA in the AOx mixture

To test selective determination of UA in the presence of other AOx compounds, a series of binary mixtures were prepared and the experiments were conducted as described in Section 2.7. Here, the CUPRAC absorbance values obtained after UOx@MNPs treatment were expressed as $A_{UOx\text{-}CUPRAC}$ and the absorbance values without UOx treatment was shown as A_{CUPRAC}. The difference between two values, *i.e.* (A_{CUPRAC} - $A_{UOx\text{-}CUPRAC}$) symbolized as ΔA, was used to calculate UA concentration. For different AOx mixtures, A_{CUPRAC}, $A_{UOx\text{-}CUPRAC}$, ΔA, concentration of UA added to the mixture (theoretical) and that calculated by using ΔA values (experimental) were shown with relative errors in Table S2.

Table S2. A_{CUPRAC}, $A_{UOx\text{-}CUPRAC}$, ΔA values, theoretical (expected) UA concentration, experimental UA concentration and relative errors for different AOx mixtures

AOx / Mixture	A_{CUPRAC}	*$A_{UOx\text{-}CUPRAC}$	ΔA	µM UA (exp.)	µM UA (theo.)	Error, %
21.7 µM UA	0.4054	0.0003	0.4051	22.34	21.70	2.93
32.6 µM UA	0.5933	0.0108	0.5825	32.64	32.60	0.12
4.35µM CFA	0.1738	-	-	-	-	-
6.52 µM CFA	0.2719	-	-	-	-	-
4.35µM QR	0.2333	-	-	-	-	-
6.52µM QR	0.3836	-	-	-	-	-
6.52µM GA	0.2147	-	-	-	-	-
8.70 µM GA	0.3063	-	-	-	-	-
4.35µM CAT	0.2267	-	-	-	-	-
6.52 µM CAT	0.3452	-	-	-	-	-
6.52 µM FA	0.1486	-	-	-	-	-
10.9 µM FA	0.2545	-	-	-	-	-
2.17 µM TR	0.2979	-	-	-	-	-
4.35 µM TR	0.5979	-	-	-	-	-
43.5 µM CYS	0.3098	-	-	-	-	-
43.5 µM NAC	0.3075	-	-	-	-	-
43.5 µM GSH	0.4930	-	-	-	-	-
21.7 µM UA + 4.35µM CFA	0.5672	0.1821	0.3851	21.17	21.70	-2.43
21.7 µM UA + 6.52µM CFA	0.6548	0.2701	0.3847	21.15	21.70	-2.53
21.7 µM UA + 4.35µM QR	0.6601	0.2840	0.3761	20.65	21.70	-4.84
21.7 µM UA + 6.52µM QR	0.7994	0.4010	0.3984	21.95	21.70	1.13
21.7 µM UA + 6.52µM GA	0.6294	0.2450	0.3844	21.13	21.70	-2.61
21.7 µM UA + 8.70µM GA	0.7252	0.3477	0.3775	20.73	21.70	-4.46
21.7 µM UA + 4.35µM CAT	0.6444	0.2619	0.3825	21.02	21.70	-3.12

21.7 μM UA + 6.52μM CAT	0.7705	0.3734	0.3971	21.87	21.70	0.79
32.6 μM UA + 4.35μM CAT	0.8012	0.2467	0.5545	31.01	32.60	-4.87
32.6 μM UA + 6.52μM CAT	0.9227	0.3655	0.5572	31.17	32.60	-4.38
21.7 μM UA + 6.52μM FA	0.5688	0.1915	0.3773	20.72	21.70	-4.51
21.7 μM UA + 10.9μM FA	0.6487	0.2684	0.3803	20.89	21.70	-3.71
21.7 μM UA + 2.17 μM TR	0.6965	0.3030	0.3935	21.66	21.70	-0.18
21.7 μM UA + 4.35 μM TR	0.9832	0.5955	0.3877	21.32	21.70	-1.73
21.7 μM UA + 43.5 μM CYS	0.7290	0.3241	0.4049	22.32	21.70	2.87
32.6 μM UA + 43.5 μM CYS	0.8854	0.2969	0.5885	32.99	32.60	1.19
21.7 μM UA + 43.5μM NAC	0.7149	0.3134	0.4015	22.13	21.70	1.96
21.7 μM UA + 43.5μM GSH	0.9010	0.5055	0.3955	21.78	21.70	0.36

* Since the absorbance values ($A_{UOx\text{-}CUPRAC}$) obtained when the CUPRAC method is applied to antioxidants containing only phenolic and thiol groups in the presence of immobilized UOx were almost the same as the absorbance values (A_{CUPRAC}) obtained by direct application of the CUPRAC method, they were not given in the table.

As described in Section S.I.3, UA determination in the presence of some serum AOxs and experimental UA concentrations were calculated as stated above. The obtained results were collected in Table S3.

Table S3. A_{CUPRAC}, $A_{UOx\text{-}CUPRAC}$, ΔA values, theoretical UA concentration, experimental UA concentration and relative errors for different serum AOx mixtures

**Serum AOx**	A_{CUPRAC}	*$A_{UOx\text{-}CUPRAC}$	**ΔA**	**μM UA (exp.)**	**μM UA (theo.)**	**Error, %**
21.7 μM UA	0.4087	0.0401	0.3686	20.73	21.70	-4.47
4.35 μM Bil	0.3077	-	-	-	-	-
17.4 μM AA	0.2721	-	-	-	-	-
21.7 μM GSH	0.2672	-	-	-	-	-
21.7 μM UA + 4.35 μM Bil	0.6999	0.3309	0.3690	20.76	21.70	-4.35
21.7 μM UA + 4.35 μM Bil +17.4 μM AA +21.7 μM GSH	1.2654	0.8573	0.4081	23.24	22.70	2.40

S.II.5. Chromatographic UA determination

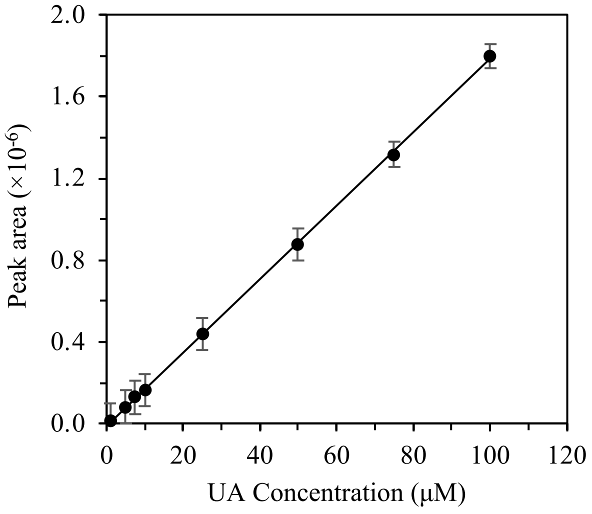

Figure S7. The calibration graph between UA concentration and HPLC peak areas.

S.II.6. Application of the proposed GOx@MNPs method to real samples

The standard addition test results were also given in Table S4.

Table S4. Recovery values obtained as a result of Glu standard addition to the FBS sample

Sample	Standard addition (µM)	Found (µM)	Recovery, %
50 µL FBS	-	48.5	-
	22.2	71.0	101.4
	33.3	82.2	101.2

As can be concluded from the quantitative recovery values in Table 3, the proposed method can be applied to Glu determination in complex samples

S.II.7. Determination of TAC and UA-free TAC in FBS by the proposed UOx@MNPs method

FBS was used as the real sample to determine UA and UA-free TAC with the developed method. The total TAC was calculated in AA equivalent units by applying the CUPRAC method to FBS and UA-added samples. Also, for UA-free TAC, the CUPRAC method was applied after the samples were treated with UOx@MNPs. The obtained results were tabulated in Table S5.

Table S5. TAC and UA-free TAC in FBS

Sample	TAC (AA Eq.) µM	UA-Free TAC (AA Eq.) µM
0.5 mL FBS	40.19	27.20
10.9 µM UA	13.14	-
21.7 µM UA	25.38	-
0.5 mL FBS + 10.9 µM UA	51.14	27.49
0.5 mL FBS + 21.7 µM UA	62.38	27.98

To compare the obtained results, the UA determination in FBS directly and in the spiked samples were analyzed by HPLC method.

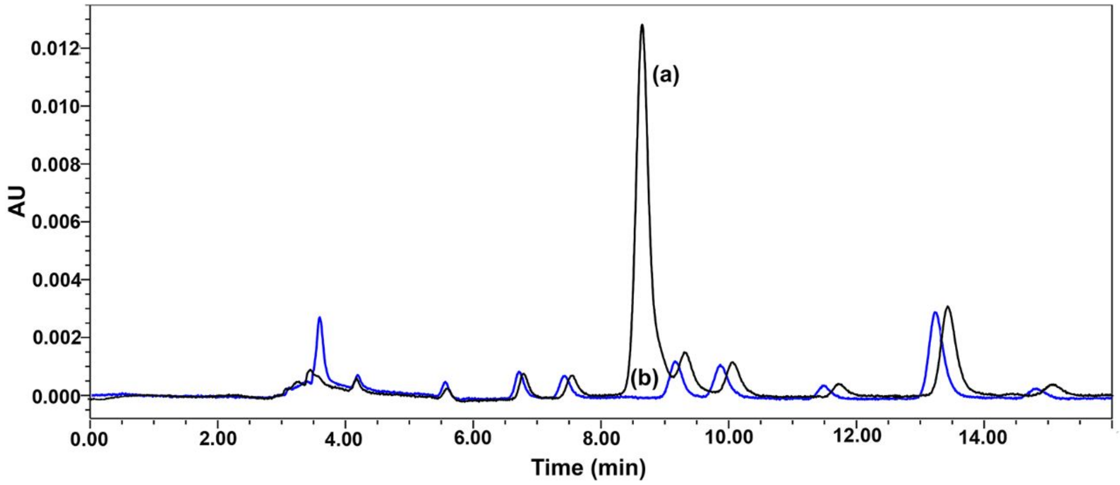

Figure S8. Chromatograms for FBS sample (a) obtained directly and (b) after UOx@MNPs treatment

As can be seen from Figure S8, UA was completely decomposed after UOx@MNPs treatment. The large peak seen in chromatogram (a) with an approximate retention time of 8.5 min was non-existent in chromatogram (b).

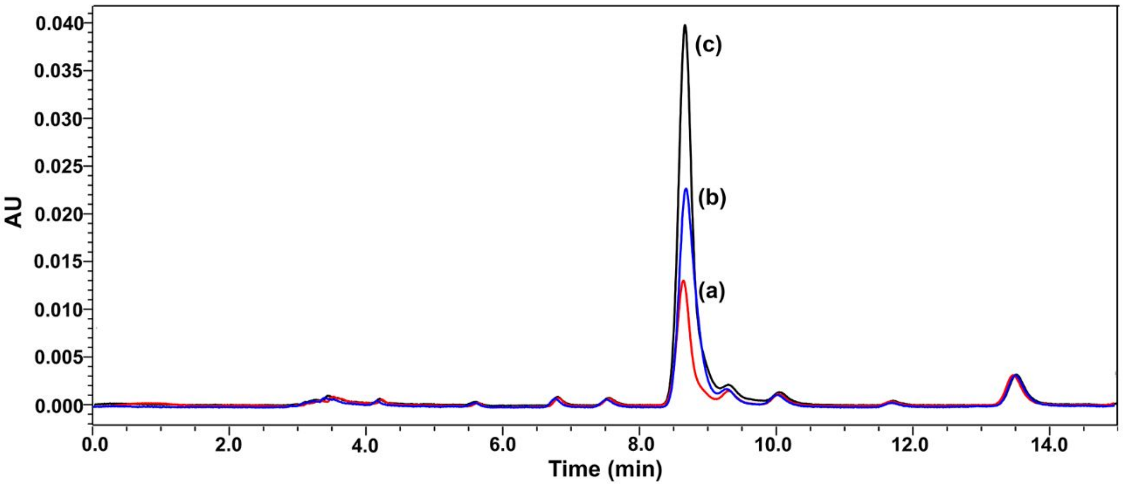

Figure S9. Chromatograms obtained for (a) FBS alone, (b) FBS + 10.9 μM UA and (c) FBS + 21.7 μM UA

The recovery values for spiked FBS samples were calculated by the proposed UOx@MNPs method and standard HPLC method.

Table S6. UA in FBS and recovery (%) values calculated with the use of UOx@MNPs method and standard HPLC method

		**The proposed UOx@MNPs method**		**HPLC method**	
**Sample**	**Final conc. of UA (in μM) added to the FBS**	**Calculated UA conc. (in μM)**	**Recovery, %**	**Calculated UA conc. (in μM)**	**Recovery, %**
0.5 mL FBS	-	10.42	-	10.84	-
	10.9	21.11	98.07	21.97	102.11
	21.7	31.78	98.43	33.31	103.55

Recovery, % values were calculated by the equation:

Recovery, % = [(Experimentally found UA conc. – UA conc. added) / UA conc. added] ×100

S.II.8. Investigation of enzyme kinetics

The results obtained by the classical UA monitoring method depending on the absorbance measurement at 290 nm for determination of the K_m and V_{max} values were given in Figure S10 and S11.

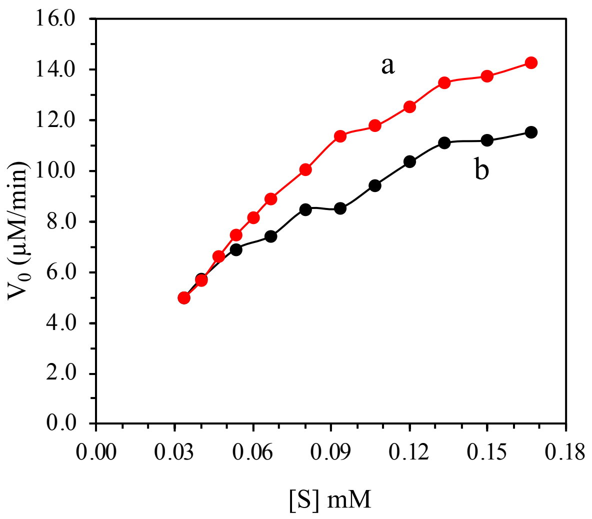

Figure S10. Michaelis-Menten plot of UA substrate for free (a) and immobilized (b) enzyme

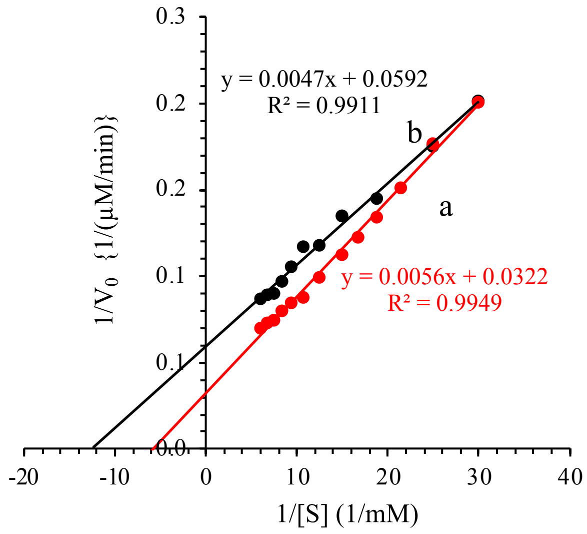

Figure S11. Lineweaver-Burk plot of UA substrate for free (a) and immobilized enzyme (b)

S.II.9. Investigation of stability and reusability of MNPs-attached enzymes

The results obtained by the experiments explained in the Section S.I.11 were given in the below for reusability (Table S7) and stability during the days (Figure S12)

Table S7. CUPRAC absorbance values obtained for two different concentrations of analytes as a result of method application by using the same enzyme-immobilized MNPs for five times consecutively.

	Measured A_{CUPRAC} for different UA concentrations(*)		Measured A_{CUPRAC} for different Glu concentrations		Measured A_{CUPRAC} for different ChCl concentrations	
**Number of reuses**	**32.6 µM UA**	**65.2 µM UA**	**33.3 µM Glu**	**88.9 µM Glu**	**11.1 µM ChCl**	**33.3 µM ChCl**
**Repeat 1**	0.0212	0.0300	0.3423	0.8284	0.3296	0.9688
**Repeat 2**	0.0238	0.0477	0.3412	0.8246	0.3281	0.9595
**Repeat 3**	0.0250	0.0533	0.3408	0.8220	0.3194	0.9417
**Repeat 4**	0.0250	0.0849	0.3383	0.8097	0.3068	0.8917
**Repeat 5**	0.0252	0.1073	0.3257	0.7879	0.3016	0.8681

(*)CUPRAC assay was applied in the presence of an additional catalase enzyme

Here it should be noted that since catalase was added to the reaction medium for UA determinations, the generated H_2O_2 equivalent to UA was decomposed and the measured absorbance values were close to zero. On the other hand, for two other analytes, namely Glu and ChCl, the absorbance values measured by CUPRAC method comes from H_2O_2 equivalent to the tested analytes.

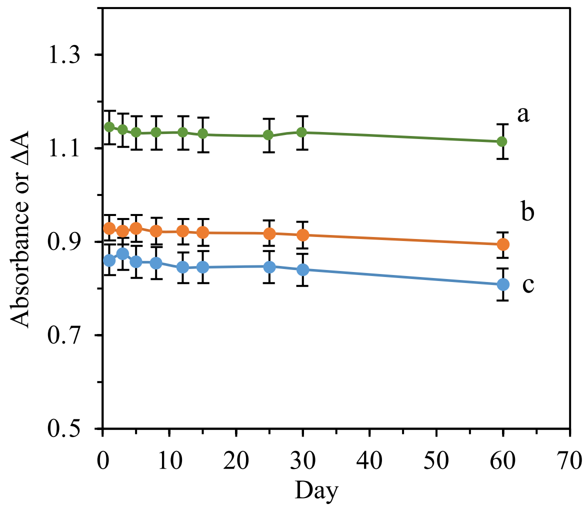

Figure S12. Absorbance (or ΔA for UA) values read at different time periods (up to 60 days) in the presence of 65.2 µM UA (a), 100 µM glucose (b) and 33.3 µM ChCl (c) using immobilized

REFERENCES

(1) Apak, R.; Güçlü, K.; Özyürek, M.; Karademir, S. E. Novel total antioxidant capacity index for dietary polyphenols and vitamins C and E, using their Cupric Ion Reducing capability in the presence of neocuproine: CUPRAC Method. *J. Agric. Food Chem.* **2004**, *52* (26), 7970–7981. https://doi.org/10.1021/JF048741X.

(2) Çekiç, S. D.; Başkan, K. S.; Tütem, E.; Apak, R. Modified Cupric Reducing Antioxidant capacity (CUPRAC) assay for measuring the antioxidant capacities of thiol-containing proteins in admixture with polyphenols. *Talanta* **2009**, *79* (2), 344–351. https://doi.org/10.1016/J.TALANTA.2009.03.061.

(3) George, S. K.; Dipu, M. T.; Mehra, U. R.; Singh, P.; Verma, A. K.; Ramgaokar, J. S. Improved HPLC method for the simultaneous determination of allantoin, uric acid and creatinine in cattle urine. *J. Chromatogr. B* **2006**, *832* (1), 134–137. https://doi.org/10.1016/J.JCHROMB.2005.10.051.

(4) Zhang, L.; Yin, S.; Hou, J.; Zhang, W.; Huang, H.; Li, Y.; Yu, C. Detection of choline and hydrogen peroxide in infant formula milk powder with near infrared upconverting luminescent nanoparticles. *Food Chem.* **2019**, *270*, 415–419. https://doi.org/10.1016/J.FOODCHEM.2018.07.128.

(5) Trinder, P. Determination of glucose in blood using glucose oxidase with an alternative oxygen acceptor. *Ann. Clin. Biochem.* **2016**, *6* (1), 24–27. https://doi.org/10.1177/000456326900600108.

(6) Wei, H.; Wang, E. Fe3O4 Magnetic Nanoparticles as Peroxidase Mimetics and Their Applications in H2O2 and Glucose Detection. *Anal. Chem.* **2008**, *80* (6), 2250–2254. https://doi.org/10.1021/AC702203F.

(7) Su, L.; Feng, J.; Zhou, X.; Ren, C.; Li, H.; Chen, X. Colorimetric detection of urine glucose based $ZnFe_2O_4$ magnetic nanoparticles. *Anal. Chem.* **2012**, *84* (13), 5753–5758. https://doi.org/10.1021/AC300939Z.

(8) Zhang, Y.; Zhang, Y.; Yang, C.; Ma, C.; Zhang, M.; Tang, J. Facile Immobilization of glucose oxidase with $Cu_3(PO_4)_2{\cdot}3H_2O$ for glucose biosensing via smartphone. *Colloids Surfaces B Biointerfaces* **2022**, *210*, 112259. https://doi.org/10.1016/J.COLSURFB.2021.112259.

(9) Nikzad, N.; Karami, Z. Label-Free colorimetric sensor for sensitive detection of choline

based on DNAzyme-choline oxidase coupling. *Int. J. Biol. Macromol.* **2018**, *115*, 1241–1248. https://doi.org/10.1016/J.IJBIOMAC.2018.04.077.

(10) Bhargava, A. K.; Lal, H.; Pundir, C. S. Discrete analysis of serum uric acid with immobilized uricase and peroxidase. *J. Biochem. Biophys. Methods* **1999**, *39* (3), 125–136. https://doi.org/10.1016/S0165-022X(99)00007-X.

(11) Chauhan, N.; Preeti; Pinky; Pundir, C. S. Covalent immobilization of uricase inside a plastic vial for uric acid determination in serum and urine. *Anal. Sci.* **2014**, *30* (4), 501–506. https://doi.org/10.2116/ANALSCI.30.501.

(12) Chi, X.; Cheng, Q.; Yang, K.; Geng, X.; Liang, Y.; Tao, J.; Wang, Z. Fabrication of a novel nano-biosensor for efficient colorimetric determination of uric acid. *Appl. Nanosci.* **2022**, *12* (7), 2255–2264. https://doi.org/10.1007/S13204-022-02498-3.

(13) Wang, X.; Tang, C. L.; Liu, J. J.; Zhang, H. Z.; Wang, J. Ultra-small CuS nanoparticles as peroxidase mimetics for sensitive and colorimetric detection of uric acid in human serum. *Chinese J. Anal. Chem.* **2018**, *46* (5), e1825–e1831. https://doi.org/10.1016/S1872-2040(17)61083-1.

(14) Zhao, H.; Wang, Z.; Jiao, X.; Zhang, L.; Lv, Y. Uricase-based highly sensitive and selective spectrophotometric determination of uric acid using BSA-stabilized Au nanoclusters as artificial enzyme. *Spectrosc. Lett.* **2012**, *45* (7), 511–519. https://doi.org/10.1080/00387010.2011.649440.

(15) Jin, D.; Seo, M. H.; Huy, B. T.; Pham, Q. T.; Conte, M. L.; Thangadurai, D.; Lee, Y. I. Quantitative determination of uric acid using CdTe nanoparticles as Fluorescence probes. *Biosens. Bioelectron.* **2016**, *77*, 359–365. https://doi.org/10.1016/J.BIOS.2015.09.057.
